# Quantum annealing with all-to-all connected nonlinear oscillators

**DOI:** 10.1038/ncomms15785

**Published:** 2017-06-08

**Authors:** Shruti Puri, Christian Kraglund Andersen, Arne L. Grimsmo, Alexandre Blais

**Affiliations:** 1 Institut quantique and Départment de Physique, Université de Sherbrooke, Sherbrooke, Québec, Canada J1K 2R1; 2 Department of Physics and Astronomy, Aarhus University, DK-8000 Aarhus, Denmark; 3 Canadian Institute for Advanced Research, Toronto MG5 1N1, Canada

## Abstract

Quantum annealing aims at solving combinatorial optimization problems mapped to Ising interactions between quantum spins. Here, with the objective of developing a noise-resilient annealer, we propose a paradigm for quantum annealing with a scalable network of two-photon-driven Kerr-nonlinear resonators. Each resonator encodes an Ising spin in a robust degenerate subspace formed by two coherent states of opposite phases. A fully connected optimization problem is mapped to local fields driving the resonators, which are connected with only local four-body interactions. We describe an adiabatic annealing protocol in this system and analyse its performance in the presence of photon loss. Numerical simulations indicate substantial resilience to this noise channel, leading to a high success probability for quantum annealing. Finally, we propose a realistic circuit QED implementation of this promising platform for implementing a large-scale quantum Ising machine.

## Abstract (web summary)

Quantum annealing aims at solving optimization problems mapped to Ising interactions between neighbouring spins, but it is crucial to implement it in a noise-resilient way. Here the authors propose a network of all-to-all connected driven Kerr-nonlinear resonators, which exhibits high success probabilities.

Many hard combinatorial optimization problems arising in diverse areas such as physics, chemistry, biology and social science^1^,^2^,^3^,^4^ can be mapped onto finding the ground state of an Ising Hamiltonian. This problem, referred to as the Ising problem, is in general NP-hard^5^. Quantum annealing, based on adiabatic quantum computing (AQC)^6^,^7^, aims to find solutions to the Ising problem, with the hope of a significant speedup over classical algorithms. In AQC, a system is evolved slowly from the non-degenerate ground state of a trivial initial Hamiltonian to that of a final Hamiltonian encoding a computational problem. During the time-evolution, the energy spectrum of the system changes and, for the adiabatic condition to be satisfied, the evolution must be slow compared to the inverse minimum energy gap between the instantaneous ground state and the excited states. The scaling behaviour of the gap with problem size, thus, determines the efficiency of the adiabatic annealing algorithm.

In order to perform quantum annealing, the Ising spins are mapped to two levels of a quantum system, that is, a qubit, and the optimization problem is encoded in the interactions between these qubits. Adiabatic optimization with a variety of physical realizations such as nuclear magnetic spins^8^ and superconducting qubits^9^,^10^ has been demonstrated. However, despite great efforts, whether these systems are able to solve large problems in the presence of noise remains an open question^11^. As a consequence, it is imperative to search for implementations with improved resilience to noise.

A general Ising problem is defined on a fully connected graph of Ising spins. However, efficient embedding of large problems with such long-range interactions is a challenge because physical systems more naturally realize local connectivity. In one approach, a fully connected graph of Ising spins is embedded in a so-called Chimera graph^12^,^13^. Alternatively, a more recent embedding scheme was proposed by Lechner, Hauke and Zoller (LHZ)^14^ in which *N* logical Ising spins are encoded in *M*=*N*(*N*−1)/2 physical spins with *M*−*N*+1 constraints. Each physical spin represents the relative configuration of a pair of logical spins. An all-to-all connected Ising problem in the logical spins is realized by mapping the logical couplings onto local fields acting on the physical spins and a problem-independent four-body coupling to enforce the constraints. This simple design requires only precise control of local fields, making it attractive for scaling to large problem sizes.

In the following, we present a physical platform for quantum annealing that is both scalable and shows robustness to noise; here we propose to encode the Ising problem in a network of two-photon-driven Kerr-nonlinear resonators (KNR). In our scheme, a single Ising spin is mapped to two coherent states with opposite phases, which constitute a two-fold degenerate eigenspace of the two-photon-driven KNR in the rotating frame of the drive^15^. Here we propose to realize quantum adiabatic algorithms by encoding a quantum spin in quasi-orthogonal coherent states. The dominant source of error in this system is single-photon loss from the resonators. However, since coherent states are invariant under the action of the photon jump operator, the encoded Ising spin is stabilized against bit flips. We describe a circuit QED implementation of a quantum annealing platform, where a fully connected graph of Ising spins is embedded using the LHZ scheme, relying on effective local magnetic fields and four-body coupling between KNRs. The adiabatic optimization is carried out by initializing the resonators to vacuum, and varying only single-site drives to adiabatically evolve the system to the ground state of the embedded Ising problem. This realization allows us to encode arbitrary Ising problems with no restriction on connectivity, or on the signs and amplitudes of the spin–spin couplings.

Encoding Ising spins in the phase of coherent states has previously been explored in the context of classical Ising machines^16^,^17^,^18^,^19^,^20^,^21^. The quantum case was considered in ref. 22. However, this previous study focussed on idealized quantum systems without noise analysis and did not consider practical implementations of these ideas. In contrast, our analysis considers the performance of quantum annealing in the presence of single-photon loss, by far the dominant loss mechanism. Crucially, we numerically demonstrate that the probability for the system to jump from the instantaneous ground state to one of its excited states due to photon loss during the adiabatic protocol is greatly suppressed as compared to conventional qubit implementations with equal noise strengths. This resilience to the detrimental effects of photon loss leads to high success probabilities in finding the optimal solution to optimization problems mapped on two-photon-driven KNRs. This noise resilience, in combination with simple initialization and final state detection by homodyne measurement of the resonators' field amplitudes, opens the door to realizing a large-scale quantum annealer with favourable noise resistance.

## Results

### Adiabatic protocol for quantum annealing

Quantum annealing is executed with the time-dependent Hamiltonian


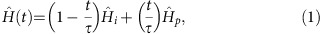


where 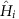 is the initial trivial Hamiltonian whose ground state is known, and 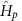 is the final Hamiltonian at *t*=*τ* which encodes an Ising spin problem: 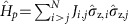. Here, 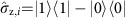 is the Pauli-*z* matrix for the *i*th spin and *J*_*i*,*j*_ is the interaction strength between the *i*th and *j*th spin. Crucially, the initial and final Hamiltonian do not commute. For simplicity, we have assumed a linear time dependence, but more complex annealing schedules can be used. The system, initialized to the ground state of 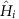, adiabatically evolves to the ground state of the problem Hamiltonian, 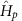, at time 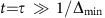, where Δ_min_ is the minimum energy gap^6^.

### Single spin in a two-photon-driven Kerr-nonlinear resonator

**Figure 1:**
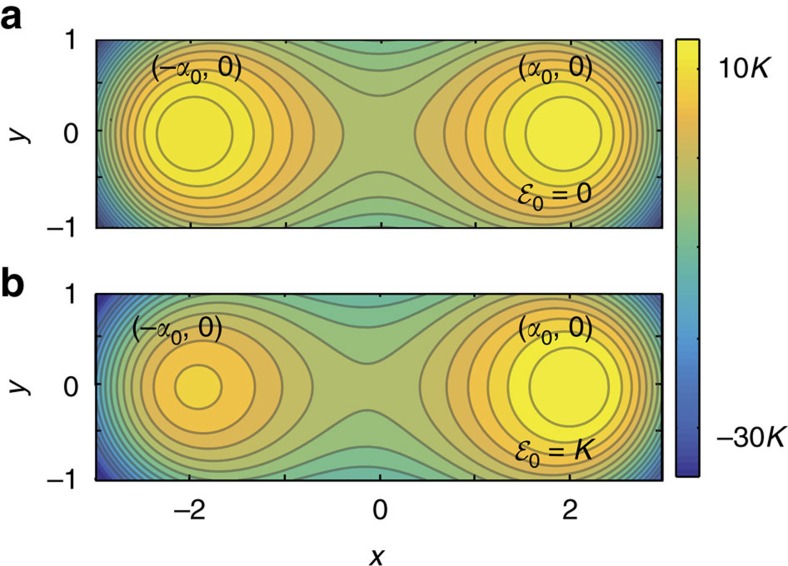
Contour plot of the metapotential. Metapotential corresponding to 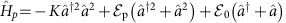, where *K* is the Kerr-nonlinearity, 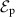 and 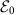 are the strengths of the two-photon and single-photon drive respectively, with 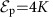 and (**a**) 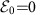, (**b**) 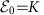. The metapotentials, shown in the units of the Kerr-nonlinearity *K*, are characterized by (**a**) two peaks of equal heights corresponding to the degenerate states 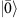 and 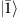, and (**b**) two peaks of different heights, indicating lifting of degeneracy between the encoded spin states 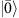 and 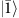.

The Hamiltonian of a two-photon driven KNR in a frame rotating at the drive frequency is given by 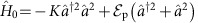, where *K* is the Kerr-nonlinearity and 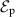 the strength of the two-photon drive. In a KNR, the coherent states 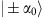, which are eigenstates of the photon annihilation operator, are stabilized by the two-photon drive with 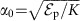 (ref. 15). This statement can be visualized more intuitively by considering the metapotential obtained by replacing the operators 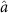 and 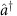 with the complex classical variables *x*+*iy* and *x*−*iy* in the expression for 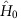 (ref. 23). As shown in Fig. 1a, this metapotential is an inverted double well with two peaks of equal height at (±*α*_0_, 0), corresponding to two stable points (see Supplementary Note 1). This is consistent with the quantum picture according to which the coherent states 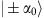 are two degenerate eigenstates of 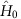 with eigenenergy 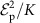 (ref. 15) (see Methods). Taking advantage of this well-defined two-state subspace, we choose to encode an Ising spin 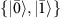 in the stable states 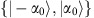. Importantly, this mapping is robust against single-photon loss from the resonator when the rate of single-photon loss is small 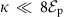, a condition than can readily be realized in superconducting circuits^15^. Moreover, the photon jump operator 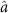 leaves the coherent states invariant 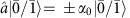. As a result, if the amplitude *α*_0_ is large such that 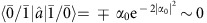, a single-photon loss does not lead to a spin-flip error.

Having defined the spin subspace, we now discuss the realization of a problem Hamiltonian in this system. As an illustrative example, we first address the trivial problem of finding the ground state of a single spin in a magnetic field. Consider the Hamiltonian of a two-photon-driven KNR with an additional weak single-photon drive of amplitude 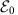, 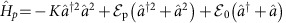. As illustrated in Fig. 1b for small 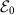, under this drive, the two peaks located at (±*α*_0_, 0) in the metapotential associated to 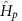 are now of unequal height with the peak at (−*α*_0_, 0) lower than the one at (*α*_0_, 0) if 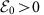, and vice versa for 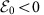. These two states remain stable, but have different energies, indicating that the single-photon drive induces an effective magnetic field on the Ising spins 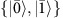. Indeed, a full quantum analysis shows that if 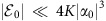, then 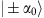 remain the eigenstates of 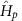 but their degeneracy is lifted by 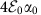 (ref. 15). In other words, in the spin subspace, 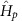 can be expressed as 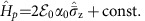, with 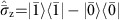. This is the required problem Hamiltonian for a single spin in a magnetic field. This simple observation will play an essential role in the implementation of the LHZ scheme discussed below. Note that for larger 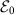, the eigenstates can deviate from coherent states (see Supplementary Note 2). Choosing 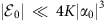, however, ensures that 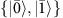 are indeed coherent states to an excellent approximation, such that 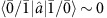 and the encoded subspace remains well protected from the photon loss channel.

Following equation (1), we require an initial Hamiltonian which does not commute with the final problem Hamiltonian and which has a simple non-degenerate ground state. This is achieved by introducing a finite detuning *δ*_0_>0 between the drives and resonator frequency. In a frame rotating at the frequency of the drives, the initial Hamiltonian is chosen as 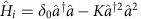 with *δ*_0_<*K*. This choice of initial Hamiltonian generates large phase fluctuations that helps maximize quantum tunnelling to states with well-defined phase at the final stages of the adiabatic evolution. In this frame, the ground and first excited states are the vacuum 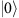 and single-photon Fock state 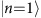, respectively, and which are separated by an energy gap *δ*_0_. If a photon is lost from the resonator, the excited state 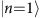 decays to the ground state 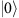 which, on the other hand, is invariant under photon loss. Since it is simple to prepare in the superconducting circuit implementation that we consider below, the vacuum state is a natural choice for the initial state.

**Figure 2:**
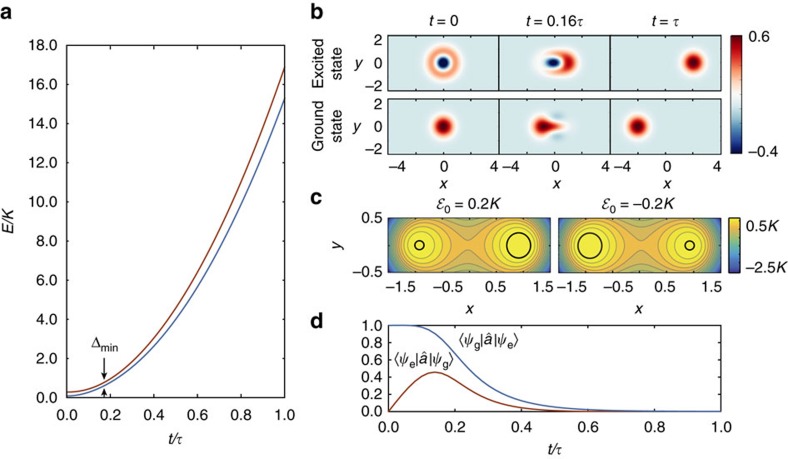
Adiabatic protocol with single spin. (**a**) Change of the energy of the ground and first excited state as a function of time in a single resonator for 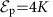, 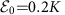 and *δ*_0_=0.2*K*. The minimum energy gap is also shown with Δ_min_=0.16*K*. (**b**) The Wigner function of the KNR state at three different times when initialized to either the excited 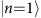 or (vacuum) ground state 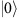, respectively. (**c**) Metapotential corresponding to 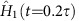 with 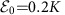 and 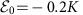 showing two peaks of unequal height. The lower peak (corresponding to the ground state) is circular, whereas the higher one (corresponding to the excited state) is deformed as highlighted by black circles. (**d**) Transition matrix elements between the ground 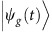 and excited states 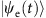 in the event of a photon jump during the adiabatic protocol.

The time-dependent Hamiltonian required for the adiabatic computation can be realized by slowly varying the two- and single-photon drive strengths and detuning so that 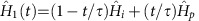, realizing equation (1) for a single-spin. Note that the form of 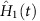 conveniently ensures that the nonlinear Kerr term is time-independent. The time-dependent detuning is achieved by tuning the single- and two-photon drive frequencies (see Methods). By adiabatically controlling the frequency and amplitude of the drives it is possible to evolve the state of the KNR from the vacuum 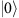 at *t*=0, to the ground state of a single Ising spin in a magnetic field at *t*=*τ*. Figure 2a shows the change of the energy landscape throughout this evolution found by numerically diagonalizing the instantaneous Hamiltonian 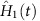 for 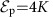, *α*_0_=2, 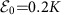 and *δ*_0_=0.2*K*. The minimum energy gap Δ_min_ is indicated. As illustrated by the Wigner functions in Fig. 2b, a resonator initialized to the vacuum state at *t*=0 evolves through highly non-classical and non-Gaussian states towards the ground state 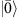 at *t*=*τ*, with 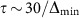 in this example. If, on the other hand, the KNR is initialized to the single-photon Fock state at *t*=0, then it evolves to the first excited state 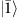 at *t*=*τ*. The average probability to reach the correct ground state is 99.9% for both 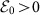 and 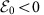. The 0.1% probability of erroneously ending in the excited state arises from non-adiabatic errors and can be reduced by increasing the evolution time. For example, for *τ*=60/Δ_min_ we find a success probability of 99.99%.

### Effect of single-photon loss

An appealing feature of this implementation is that, at the start of the adiabatic protocol, the ground (vacuum) state is invariant under single-photon loss. Similarly, at the end of the adiabatic protocol at *t*=*τ*, irrespective of the problem Hamiltonian (that is, 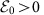 or 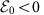) the ground state (coherent states 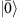 or 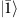) is also invariant under single-photon loss. It follows that towards the beginning and end of the protocol, photon loss will not induce errors. Moreover, we find that, even at intermediate times 0<*t*<*τ*, the ground state of 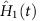 remains largely unaffected by photon loss. This can be understood intuitively from the distortion of the metapotential, as shown in Fig. 2c at *t*=0.2*τ* for the same parameters as Fig. 2a. While the metapotential still shows two peaks, the region around the lower peak (corresponding to the ground state) is a circle whereas that around the higher peak (corresponding to the excited state) is deformed. This suggests that the ground state is closer to a coherent state and, therefore, more robust to photon loss than the excited state (see also Supplementary Note 3). Quantitatively, the effect of single-photon loss is seen by numerically evaluating^24^,^25^ the transition matrix elements 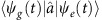, 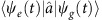 for the duration of the protocol, where 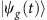 and 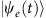 are the ground and excited state of 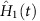 respectively. As shown in Fig. 2d, the transition from the ground to excited state is greatly suppressed throughout the whole adiabatic evolution. This asymmetry in the transition rates distinguishes AQC with two-photon-driven KNRs from implementations with qubits^26^, something that will be made even clearer below with examples.

### Two coupled spins with driven KNRs

Before going to larger lattices, consider the problem of two interacting spins embedded in a system of two linearly coupled KNRs, each driven by a two-photon drive and given by the Hamiltonian 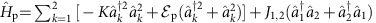. Here, *J*_1,2_ is the amplitude of the single-photon exchange coupling and, for simplicity, the two resonators are assumed to have identical parameters. For small *J*_1,2_, this Hamiltonian can be expressed in the 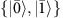 basis as the problem Hamiltonian^15^ 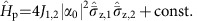 The nature of the interaction is encoded in the phase of the coupling with *J*_1,2_<0 (*J*_1,2_>0) corresponding to the ferromagnetic (anti-ferromagnetic) case. For the initial Hamiltonian, we take 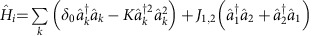 and, following equation (1), the full time-dependent Hamiltonian for the two-spin problem is 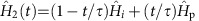. Although it is possible to tune these parameters in time, with the above form of 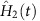, both the linear coupling and the Kerr-nonlinearity are fixed throughout the adiabatic evolution.

The ground state of 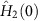 is the vacuum state if the initial detuning is greater than the single-photon exchange rate, *δ*_0_>*J*_1,2_. On the other hand, at *t*=*τ*, the two degenerate ground states for anti-ferromagnetic (ferromagnetic) coupling are 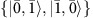 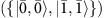. Accordingly, numerical simulations with both resonators initialized to vacuum show the coupled system to reach the entangled state 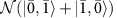 and 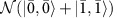, under anti-ferromagnetic and ferromagnetic coupling, respectively. In these expressions, 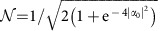 is a normalization constant. With the realistic parameters *τ*=50/Δ_min_, *δ*_0_=*K*/4, *J*_1,2_=*K*/10 and 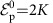, corresponding to 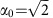, the state fidelity is 99.9%. Moreover, the probability that the system is in any one of the states 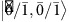 is 99.99%, showing that the evolution is to a very good approximation restricted to this computational subspace.

While the states used in this encoding are tolerant to photon loss, coherence between a superposition of those states is reduced. However the success probability (see Methods) in solving the Ising problem remains high as it depends only on the diagonal elements of the density matrix (for example, 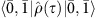). As an illustration, with the large loss rate *κ*=50/*τ*, while the fidelity of the final state to the desired superposition 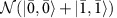 or 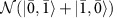 decreases to 37.6%, the average success probability of the algorithm is 75.2%.

**Figure 3:**
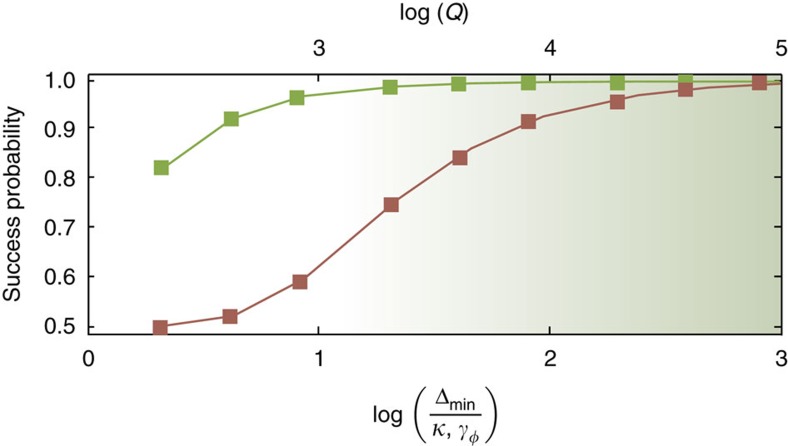
Success probability for the two coupled spins problem. Loss-rate dependence of the success probability for the two-spin adiabatic algorithm in a system of two-photon-driven KNRs with single-photon loss *κ* (green squares) and qubits with pure dephasing at rate *γ*_*φ*_ (red squares). The quality factor *Q*=*ω*_r_/*κ* is indicated on the top axis for a KNR of frequency *ω*_r_/2*π*=5 GHz.

To characterize the effect of noise, a useful figure of merit is the ratio Δ_min_/*κ* of the minimum energy gap to the loss rate. The dependence of the average success probability on this ratio is presented in Fig. 3 for the algorithm implemented using KNRs (green squares) with single-photon loss *κ* or qubits (red squares) with pure dephasing *γ*_*φ*_. In practice, the average success probability is computed by varying the loss rates at fixed Δ_min_ and *τ*=20/Δ_min_, and is averaged over all instances of the problem of two coupled spins (that is, ferromagnetic and anti-ferromagnetic). In the presence of pure dephasing, the success probability with qubits saturates to 50% for large *γ*_*φ*_. This is a consequence of the fact that the steady state of the qubits is an equal weight classical mixture of all possible computational states. On the other hand, for KNRs with a finite *κ*, the rate at which the instantaneous ground state jumps to the excited state (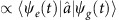) is small compared to the rate at which the instantaneous excited state jumps to the ground state (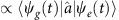). As a result, even with large single-photon loss rate, for example 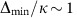, the success probability is ∼75%. Consequently, in the presence of equivalent strength noise, a two-photon-driven KNR implementation of AQC has superior performance compared to a qubit implementation (see Methods for details).

### All-to-all connected Ising problem with the LHZ scheme

The above scheme can be scaled up with pairwise linear couplings in a network of KNRs, while still requiring only single-site drives. However, unlike the above one- and two-spin examples, most optimization problems of interest require controllable long-range interactions between a large number of Ising spins. Realizing such highly non-local Hamiltonian is a challenging hardware problem, but it may be solved by embeddings such as the LHZ scheme^14^ that map the Ising problem on a graph with local interactions only. In this approach, the relative configuration of pairs of *N* logical spins is mapped to *M*=*N*(*N*−1)/2 physical spins. A pair of logical spins, in which both spins are aligned |0, 0〉 or |1, 1〉 (or anti-aligned |0, 1〉 or |1, 0〉), is mapped on the two levels of the physical spin. The coupling between the logical pairs *J*_*i*,*j*_ (*i*=1, ..*N*) is encoded in local magnetic fields on the physical spins *J*_*k*_ (*k*=1, …, *M*). For a consistent mapping, *M*−*N*+1 energy penalties in the form of four-body coupling are introduced to enforce an even number of spin-flips around any closed loop in the logical spins. It was shown in ref. 14 that a fully connected graph can then be encoded in a planar architecture with only local connectivity. The problem Hamiltonian in the physical spin basis becomes 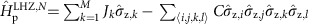, where 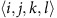 denotes the nearest-neighbour spins enforcing the constraint.

**Figure 4:**
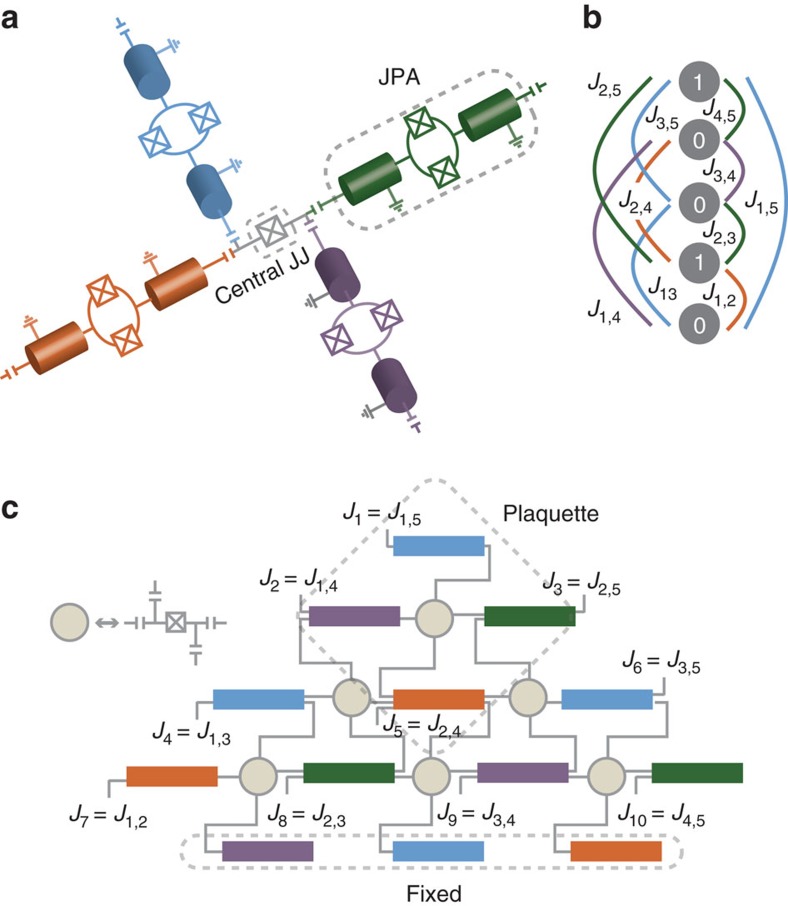
Physical realization of the LHZ scheme. (**a**) Illustration of the plaquette consisting of four JPAs coupled by a Josephson junction (JJ). The four JPAs have different frequencies (indicated by colours) and are driven by two-photon drives such that *ω*_p,*k*_+*ω*_p,*l*_=*ω*_p,*m*_+*ω*_p,*n*_. The nonlinearity of the JJ induces a four-body coupling between the KNRs. (**b**) Illustration of a fully connected Ising problem with *N*=5 logical spins. (**c**) The same problem embedded on *M*=10 physical spins and 3 fixed spins on the boundary.

We now describe a circuit QED platform implementing the LHZ scheme by embedding the physical spins in the eigenbasis 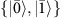 of two-photon-driven KNRs. In practice, KNRs can be realized as a superconducting microwave resonator terminated by a flux-pumped SQUID. The non-linear inductance of the SQUIDs induces a Kerr-nonlinearity, and a two-photon drive is introduced by flux-pumping at twice the resonator frequency. This is the exact same setup that is used to realize Josephson parametric amplifiers (JPAs), and we will therefore refer to this implementation of a KNR as a JPA in the following^15^,^27^,^28^,^29^. We envision a quantum annealing platform to be built with groups of four JPAs of frequencies *ω*_r,*i*_ (*i*=1, 2, 3, 4) interacting via a single Josephson junction (JJ) as illustrated in Fig. 4a. In the figure, the four different frequencies of the resonators are indicated by four different colours. To realize a time-dependent two-photon drive, the SQUID loop of each JPA is driven by a flux pump with tunable amplitude and frequency. The pump frequency is varied close to twice the resonator frequency, 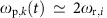 (see Methods). Additional single-photon drives, whose amplitude and frequency can be varied in time, are also applied to each of the JPAs and play the role of effective local magnetic fields. Local four-body couplings are realized through the nonlinear inductance of the central JJ (see Supplementary Note 5). Choosing *ω*_p,*k*_(*t*)+*ω*_p,*l*_(*t*)=*ω*_p,*m*_(*t*)+*ω*_p,*n*_(*t*) and taking the resonators to be detuned from each other, the central JJ induces a coupling of the form 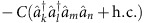 in the instantaneous rotating frame of the two-photon drives. This four-body interaction is an always-on coupling and its strength *C* is determined by the JJ nonlinearity. Such a group of four JPAs, which we will refer to as a plaquette, is the central building block of our architecture and can be scaled in the form of the triangular lattice required to implement the LHZ scheme^14^. Note that while JPAs within a plaquette have different frequencies, only four distinct JPA frequencies are required for the entire lattice as illustrated in Fig. 4c. Lastly, the LHZ scheme also requires additional *N*−2 physical spins at the boundary that are fixed to the up state and which are implemented in our scheme as JPAs stabilized in the eigenstate 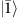 by two-photon drives. As an illustration, Fig. 4b depicts all the possible interactions in an Ising problem with *N*=5 logical spins and Fig. 4c shows the corresponding triangular network of coupled JPAs. To implement the adiabatic protocol for a general *N*-spin Ising problem with the triangular network of *M* JPAs, the time-dependent Hamiltonian in a frame where each of the JPAs rotate at the instantaneous drive frequency can be written as


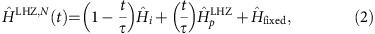


where


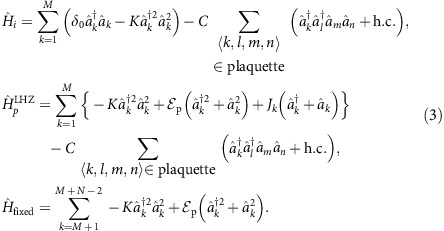


As mentioned above, *M*=*N*(*N*−1)/2 while *J*_*k*_ is the single-photon drive which induces the local effective magnetic field on the *k*th resonator and *C* is the local four-body coupling between the resonators. A final necessary component for a quantum annealing architecture is readout of the state of the physical spin. Here, this is realized by standard homodyne detection which can resolve the two coherent states 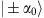 allowing the determination of the ground state configuration of the spins.

In order to demonstrate the adiabatic algorithm for a non-trivial case, we embed on a plaquette a simple three-spin frustrated Ising problem, in which the spins are anti-ferromagnetically coupled to each other, 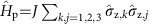 with *J*>0. This Hamiltonian has six degenerate ground states in the logical spin basis. Following the LHZ approach, a mapping of *N*=3 logical spins requires *M*=3 physical spins (in our case three JPAs) and one physical spin fixed to up state (in our case a JPA initialized to the stable eigenstate 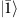). Since the physical spins 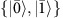 encoded in the JPAs constitute the relative alignment of the logical spins, there are three possible solutions in this basis: 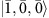, 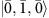 and 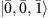. The time-dependent Hamiltonian for the adiabatic protocol then follows from equation (2) with *M*=3. The anti-ferromagnetic coupling between the logical spins is represented by the single-photon drives on each JPA with amplitude *J*_*k*_=*J*>0. At *t*=0, the ground state of this Hamiltonian is the vacuum 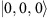. For appropriate magnitude of the four-body coupling (see Supplementary Note 4), the problem Hamiltonian can be expressed as 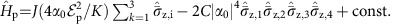 with 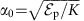. This realizes the required problem Hamiltonian in the LHZ scheme 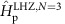.

**Figure 5:**
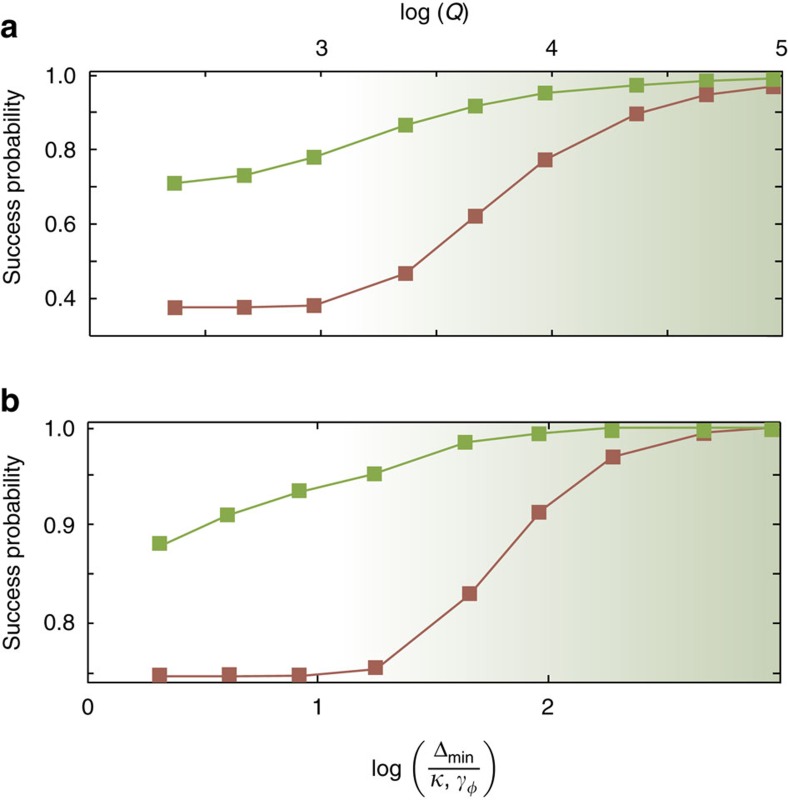
Success probability for the frustrated three-spin problem. (**a**) With LHZ encoding: Probability of successfully finding the ground state of a frustrated three-spin Ising problem by implementing the adiabatic algorithm on a plaquette of four KNRs with single-photon loss (green squares) for 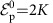, *δ*_0_=0.45*K*, *C*=0.05*K*, *J*=0.095*K*. The success probability for an implementation with qubits with pure dephasing rate *γ*_*φ*_ is also shown (red squares). The two cases are designed to have identical Δ_min_ and computation time *τ*=40/Δ_min_. The quality factor *Q*=*ω*_r_/*κ* is indicated on the top axis for a KNR of frequency *ω*_r_/2*π*=5 GHz. (**b**) Without encoding: Probability of successfully finding the ground state of a frustrated three-spin Ising problem by implementing the adiabatic algorithm on three directly coupled KNRs with single-photon loss (green squares) for 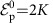, *δ*_0_=0.45*K*, *J*_*k*,*j*_=0.095*K* for *k*,*i*=1, 2, 3. Note that the local drive *J* in the embedded problem is same as the coupling *J*_*k*,*j*_ in the un-embedded one and the minimum energy gap in the un-embedded problem is twice that of the embedded problem. The success probability for an implementation with qubits without encoding and with pure dephasing is also shown (red squares).

To illustrate the performance of this protocol, we numerically simulate the evolution subjected to the Hamiltonian of equation (2) with the three resonators initialized to vacuum and the fourth initialized to the state 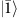. With 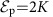, 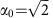, *J*=0.095*K*, *C*=0.05*K*, *τ*=40/Δ_min_ and *κ*=0, we find that the success probability to reach the ground state to be 99.3%. The reduction in fidelity arises from the non-adiabatic errors. The probability for the system to be in one of the states 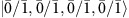 is 99.98% indicating that, with high accuracy, the final state is restricted to this subspace. Figure 5a shows the dependence of the success probability on single-photon loss rate (green). It also presents the success probability when the algorithm is implemented with qubits (red) subjected to dephasing noise (see Methods). Again, we find that, in the presence of equal strength noise, the adiabatic protocol with JPAs (or two-photon-driven KNRs) has superior performance with respect to qubits. Figure 5b also shows the success probability for the same problem but without using the LHZ embedding, that is, when the three KNRs (green) or qubits (red) are directly coupled to each other via a two-body interaction of the form 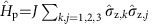 with *J*>0 (see Methods). As with embedding, the success probability with KNRs is higher than with qubits for equal strength noise. For the particular example considered here, the degeneracy of the ground state is higher in the un-embedded problem (six) than for the embedded problem (three). As a result, the likelihood to remain in one of the ground states increases and, in the presence of noise, the un-embedded problem performs slightly better than the embedded problem. These examples of simple frustrated three-spin problems demonstrate the performance of a single plaquette. Embedding of large Ising problems requires more plaquettes connected together as shown in Fig. 4. Even in such a larger lattice, each JPA is connected to only four other JPAs, making it likely that the final state remains restricted to the encoded subspace spanned by the states 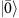, 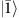.

## Discussion

We have introduced an adiabatic protocol performing quantum annealing with all-to-all connected Ising spins in a network of non-linear resonators with only local interactions. We have analysed the performances of this annealer in the presence of single-photon loss and shown that the success probability is considerably higher compared to qubits with same amount of loss. Although the implementation of the LHZ scheme has been explored here, other embeddings schemes such as minor embedding could be realized by taking advantage of single-photon exchange and the corresponding two-body couplings that it results in. A distinguishing feature of our scheme is that the spins are encoded in continuous-variable states of resonator fields. The restriction to two approximately orthogonal coherent states only happens in the late stage of the adiabatic evolution, and in general each site must be treated as a continuous variable system displaying rich physics, exemplified by non-Gaussian states, with negative-valued Wigner functions. Because the negativity of the Wigner function is directly related to classical non-simulability^30^,^31^,^32^, how this behaviour persists in the presence of photon loss with increasing problem sizes is an interesting question.

Another promising avenue to explore is how the continuous variable nature of our system influences the annealer's computational capabilities as compared to more conventional approaches based on two-level systems evolving under a transverse field Ising Hamiltonian, that is, where equation (1) is built from 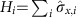 and 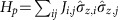 (refs 9, 33). For instance, we showed how the nature of the quantum fluctuations around the instantaneous ground and excited states leads to increased stability of the ground state. As the size of the system increases, these continuous variable states might alter the nature of phase transitions during the adiabatic evolution, something which may lead to changes in computational power^34^,^35^. It is also worth pointing out that our circuit QED implementation easily allows for adding correlated phase fluctuations given by interaction terms like 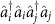 (see Supplementary Note 6). These terms do not affect the energy spectrum of the encoded problem Hamiltonian, but may modify the scaling of the minimal gap during the annealing protocol.

Yet another appealing feature that motivates further study is that the time-dependent Hamiltonian of KNRs is generically non-stoquastic in the number basis. A stoquastic Hamiltonian by definition only has real, non-positive off-diagonal entries^36^, and the Hamiltonians in this class are directly amenable to quantum Monte Carlo simulations (stoquastic Hamiltonians do not have the so-called ‘sign problem'). As an example, the transverse field Ising Hamiltonian, which is the focus of much current experimental efforts^9^,^33^, is stoquastic. In contrast, the Hamiltonian of our system has off-diagonal terms 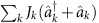 in the LHZ embedding (or 
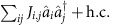
 if this embedding is not used) with problem-dependent signs (note that simply mapping 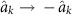 does not solve the problem due to the presence of the quartic terms equation (2)). The same is true if one considers matrix elements in the over-complete basis of coherent states. Non-stoquasticity has been linked to exponential speedups in quantum annealing^37^, and is widely believed to be necessary to gain more than constant speedup over classical devices^38^.

Ultimately, further investigation into the performance of our adiabatic protocol on larger problem sizes is warranted. Currently, the large Hilbert space size prevents numerically exact simulations with more than a few resonators. Nonetheless, the results here strongly suggest that the adiabatic protocol with two-photon-driven KNRs has excellent resistance to photon loss and thermal noise. Together with the highly non-classical physics displayed during the adiabatic evolution, this motivates the realization of a robust, scalable quantum Ising machine based on this architecture. After completing this work, we became aware of an alternative approach to quantum annealing with Kerr parametric oscillator^39^.

## Methods

### Eigen-subspace of a two-photon-driven KNR

Following ref. 15, the Hamiltonian of the two-photon-driven KNR can be expressed as


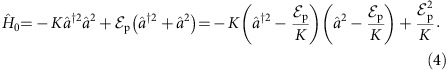


This form makes it clear that the two coherent states 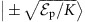, which are the eigenstates of the annihilation operator 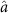, are also degenerate eigenstates of equation (4) with energy 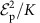.

### Time-dependent Hamiltonian in the instantaneous rotating frame

We describe the required time-dependence of the amplitude and frequency of the drives to obtain the time-dependent Hamiltonians needed for the adiabatic protocol. As an illustration, consider the example of a two-photon-driven KNR with additional single-photon drive whose Hamiltonian is written in the laboratory frame as


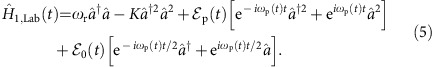


Here, *ω*_r_ is the fixed KNR frequency and *ω*_p_(*t*) is the time-dependent two-photon drive frequency. The frequency of the single-photon drive, of amplitude 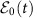, is chosen to be *ω*_p_(*t*)/2 such that it is on resonance with the two-photon drive. In a rotating frame defined by the unitary transformation 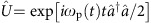, this Hamiltonian reads


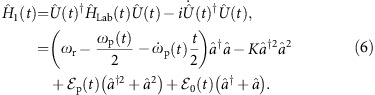


Choosing the time dependence of the drive frequency as *ω*_p_(*t*)=2*ω*_r_−2*δ*_0_(1−*t*/2*τ*), and the drive strengths as 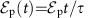 and 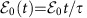, the above Hamiltonian simplifies to


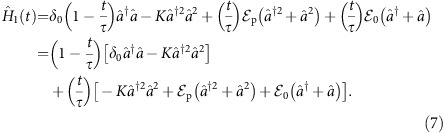


This has the standard form of a linear interpolation between an initial Hamiltonian and a problem Hamiltonian that is required to implement the adiabatic protocol.

As a second illustration, the time-dependent Hamiltonian for finding the ground state of a frustrated three-spin problem embedded on a plaquette is


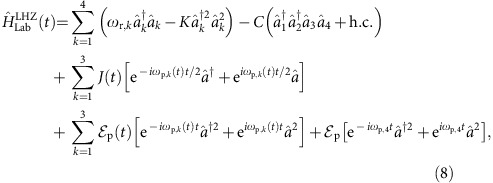


where *ω*_r,*k*_ are the fixed resonator frequencies and *ω*_p,*k*_(*t*) the time-dependent two-photon drive frequencies. The resonators labelled *k*=1, 2 and 3 are driven by time-dependent two-photon and single-photon drives of strengths 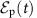, *J*(*t*) and frequency *ω*_p,*k*_(*t*), *ω*_p,*k*_(*t*)/2, respectively. On the other hand, the frequency and strength of the two-photon drive on the *k*=4 resonator is fixed. Applying the unitary 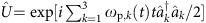 leads to the transformed Hamiltonian


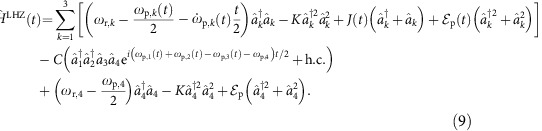


To realize equation (2) implementing the adiabatic algorithm on this plaquette, we choose the drive frequencies such that *ω*_p,*k*_(*t*)=2*ω*_r,*k*_−2*δ*_0_(1−*t*/2*τ*) and *ω*_p,4_=2*ω*_r,4_, with their sum respecting *ω*_p,1_(*t*)+*ω*_p,2_(*t*)=*ω*_p,3_(*t*)+*ω*_p,4_. Moreover, we take the time-dependent amplitudes 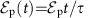 and *J*(*t*)=*Jt*/*τ*.

### Estimation of success probability:

To estimate the success probability of the adiabatic algorithm with KNRs, as shown by the green squares in Fig. 3, we numerically simulate^24^,^25^ the master equation 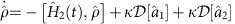, where photon loss is accounted for by the Lindbladian 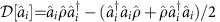. It is important to keep in mind that even though the energy gap is small in the rotating frame, the KNRs laboratory frame frequencies *ω*_r,*k*_ are by far the largest energy scale. As a result, the above standard quantum optics master equation correctly describes damping in this system^40^. Moreover, because we are working with KNR frequencies in the GHz range, as is typical with superconducting circuits, thermal fluctuations are negligible. From this master equation, the success probability can be evaluated as the probability of occupation of the correct ground state at the final time *t*=*τ*, that is, 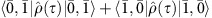 and 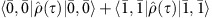 for 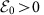 and 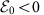, respectively.

On the other hand, the master equation used to simulate the adiabatic algorithm with qubits is 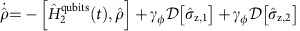 where


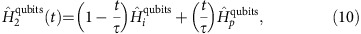



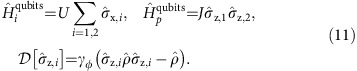


Here, 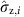 and 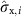 are Pauli operators in the computational basis formed by the ground 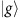 and excited state 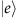 of the *i*th qubit. In these simulations, the qubits are initialized to the ground state of the initial transverse field, and the success probability (red squares in Fig. 3) is computed as the probability of occupation of the correct ground state at *t*=*τ*, that is, 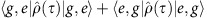 and 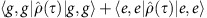 for *J*>0 and *J*<0, respectively.

Finally, to obtain the data for the resonators in Fig. 5 (green squares), the simulated master equation is 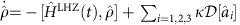 while, for qubits, it is 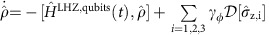. In these expressions,


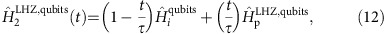



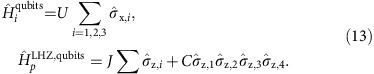


The success probability is computed as the probability of occupation of the correct ground state at *t*=*τ*, that is, 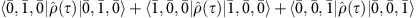 (green squares in Fig. 5) and 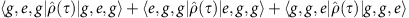 (red squares in Fig. 5).

### Two paradigms of quantum annealing

The KNR-based quantum annealer proposed in this paper is based on an adiabatic non-equilibrium evolution, where the system is subject to driven and dissipative processes. This is in stark contrast to the conventional approach to quantum annealing, where a quantum system is at all times in thermal equilibrium at very low temperature such that it stays close to the ground state, as Hamiltonian parameters are adiabatically varied^1^. It is crucial to understand the very different roles played by the bath, modelling the environment of the annealer, in these two different approaches. In the conventional approach, as long as the temperature is sufficiently low compared to the energy gap, the thermal population of the first excited state is negligible and the system is effectively in its ground state. In fact, for large gaps, the coupling to the environment typically *helps* the annealer by constantly cooling the system towards its ground state. On the other hand, the bath becomes detrimental and will typically lead to large errors as soon as 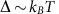. Since the gap decreases exponentially with problem size for hard problems, this is a major roadblock for conventional quantum annealing. Even for easier problems when the gap closes polynomially, it quickly becomes extremely challenging to go to large system sizes.

The type of non-equilibrium quantum annealer considered in the present paper overcomes this roadblock, but trades the difficulty for a related but different challenge. The crucial point is that although the gap is small in the rotating frame where the annealing schedule is realized, the system still probes the environment at a very high frequency. All coupling and interaction terms in the Hamiltonian are effectively small perturbations of the resonator's bare Hamiltonian 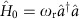, such that the energy cost of adding a thermal photon is to a very good approximation *ℏω*_r_, giving a negligible thermal population, *N*_th_=exp(−*ℏω*_r_/*k*_*B*_*T*), for typical frequencies in the 5–15 GHz range and temperatures *T*∼10 mK. This is also the justification for the master equations used when computing the success probabilities Figs 3 and 5.

Although thermal noise is no longer a bottleneck for this type of non-equilibrium quantum annealing, another challenge now arises. Since the system is not in equilibrium, the eigenstates of the rotating frame Hamiltonian are not global eigenstates of the total system, including the bath, and the interaction with the bath therefore does not generically drive the system towards the rotating-frame ground state, even at zero temperature (see Supplementary Note 3). This leads to *local* dephasing noise for the KNR implementation due to resonator photon loss. We emphasize that when comparing to a qubit implementation in Figs 3 and 5, we are comparing to an analogous implementation where the qubits are also only subjected to local dephasing noise, as opposed to thermal noise due to a small gap. This allows a fair comparison of two different physical systems used to realize the Ising spins under equal noise strength, and applies for example to realizations of the type proposed in ref. 26. How non-equilibrium quantum annealing compares to conventional equilibrium quantum annealing more generally is to the best of our knowledge an open problem, and an interesting and important avenue for future research.

### Realization of four-body coupling

The physical realization of the four-body coupling is described here and more details can be found in Supplementary Notes 6 and 8. The photon annihilation operators of the four KNRs each of which are driven with a two-photon drive are denoted by 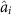, with *i*=1, 2, 3, 4. These resonators are capacitively coupled to a central JJ described by the annihilation operator 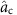 and of energy 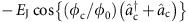. In this expression, *E*_J_ is the Josephson energy, *φ*_0_=*ℏ*/2*e* is the reduced flux quantum and *φ*_c_ is the standard deviation of the zero-point flux fluctuation for the junction mode. The coupling strength *g*_*i*_ between the resonators and the junction is smaller than the detuning between them Δ_*i*_, 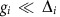. In this dispersive, limit the mode of the junction becomes dressed with the resonator modes 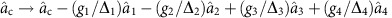 and the Josephson energy becomes


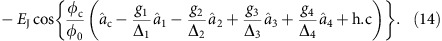


The fourth-order expansion of the cosine leads to a coupling 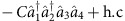, where 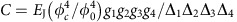. In the rotating frame of the drive, this coupling becomes resonant when the frequencies are chosen such that *ω*_p,1_(*t*)+*ω*_p,2_(*t*)=*ω*_p,3_(*t*)+*ω*_p,4_(*t*). In addition to the above four-body coupling, cross-Kerr terms of the form 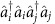 are also resonant. As shown in Supplementary Note 7, these terms do not affect the success probability of the algorithm. The strength of the coupling can be estimated with typical parameters *E*_*J*_/2*π*=600 GHz, *φ*_*c*_=0.12*φ*_0_, 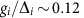, resulting in *C*/2*π*=63 KHz. For a typical strength of Kerr-nonlinearity *K*/2*π*=600 KHz, this leads to 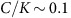.

### Data availability

The data that support the findings of this study are available from the corresponding author on reasonable request.

## Additional information

**How to cite this article:** Puri, S. *et al*. Quantum annealing with all-to-all connected nonlinear oscillators. *Nat. Commun.* **8,** 15785 doi: 10.1038/ncomms15785 (2017).

**Publisher's note**: Springer Nature remains neutral with regard to jurisdictional claims in published maps and institutional affiliations.

## Supplementary Material

Supplementary Information Supplementary figures, supplementary notes and supplementary references.
