# Supplementary material for: Quantum annealing with all-to-all connected nonlinear oscillators
Source: Nat Commun. 2017 Jun 8;8:15785. doi: 10.1038/ncomms15785 (PMC5472748; doi:10.1038/ncomms15785)
Supplement: Supplementary Information — Supplementary figures, supplementary notes and supplementary references. [file ncomms15785-s1.pdf]

SUPPLEMENTARY NOTE 1: STABILITY ANALYSIS OF THE META-POTENTIAL FOR A SINGLE TWO-PHOTON DRIVEN KNR

The coherent states $|\pm\alpha_0\rangle$, where $\alpha_0 = \sqrt{\mathcal{E}_\mathrm{p}/K}$, are eigenstates of the two-photon driven KNR Hamiltonian [1]

$$\hat{H}_0 = -K\hat{a}^{\dagger 2}\hat{a}^2 + \mathcal{E}_\mathrm{p}(\hat{a}^{\dagger 2} + \hat{a}^2). \tag{1}$$

Following Ref. [1], we will outline a proof of this statement that will serve as the foundation for the analysis in the Supplementary Notes 2 and 3. We start by applying a displacement transformation $D(\alpha) = \exp(\alpha\hat{a}^\dagger - \alpha\hat{a})$ to $\hat{H}_0$, so that

$$\hat{H}_0' = D^\dagger(\alpha)\hat{H}_0 D(\alpha) \tag{2}$$
$$= \left[(-2K\alpha^2\alpha^* + 2\mathcal{E}_\mathrm{p}\alpha^*)\hat{a}^\dagger + \mathrm{h.c.}\right] + \left[(-K\alpha^2 + \mathcal{E}_\mathrm{p})\hat{a}^{\dagger 2} + \mathrm{h.c.}\right] - 4K|\alpha|^2\hat{a}^\dagger\hat{a} - K\hat{a}^{\dagger 2}\hat{a}^2 - (2K\alpha\hat{a}^{\dagger 2}\hat{a} + \mathrm{h.c.}). \tag{3}$$

In the above expression, we have dropped the constant term $E(\alpha) = -K|\alpha|^4 + \mathcal{E}_\mathrm{p}^*\alpha^2 + \mathcal{E}_\mathrm{p}\alpha^{*2}$ which represents a shift in energy. We choose α such that the coefficient of $\hat{a}^\dagger$ satisfies $-2K\alpha^2\alpha^* + 2\mathcal{E}_\mathrm{p}\alpha^* = 0$, which is equivalent to finding the turning points of the metapotential of Fig. 1 of the manuscript. This equation has three solutions: $(0,0), (\pm\alpha_0, 0)$ corresponding to the dip and two peaks of the inverted double-well metapotential. At $(0,0)$, the Hamiltonian in Eq. (3) represents a resonantly driven parametric amplifier. Therefore, in the absence of losses, large fluctuations make the system unstable around $(0,0)$ [2]. On the other hand, at $(\pm\alpha_0, 0)$, the Hamiltonian Eq. (3) takes the form

$$\hat{H}_0'(\alpha = \pm\alpha_0) = -4K|\alpha_0|^2\hat{a}^\dagger\hat{a} - K\hat{a}^{\dagger 2}\hat{a}^2 - (\pm 2K\alpha_0\hat{a}^{\dagger 2}\hat{a} + \mathrm{h.c.}). \tag{4}$$

With this normally ordered form, we immediately conclude that the vacuum $|0\rangle$ is an eigenstate of $\hat{H}_0'$ in the displaced frame. It immediately follows that the coherent states $|\pm\alpha_0\rangle$ are the eigenstates of the Hamiltonian $\hat{H}_0$ in the non-displaced frame. Furthermore, these are degenerate eigenstates as $E(\alpha_0) = E(-\alpha_0)$.

SUPPLEMENTARY NOTE 2: THE EIGEN-SUBSPACE IN THE PRESENCE OF SINGLE-PHOTON DRIVE

The Hamiltonian of a two-photon driven KNR with an additional single-photon drive is given by

$$\hat{H} = -K\hat{a}^\dagger\hat{a}^\dagger\hat{a}\hat{a} + \mathcal{E}_\mathrm{p}(\hat{a}^{\dagger 2} + \hat{a}^2) + \mathcal{E}_0(\hat{a}^\dagger + \hat{a}). \tag{5}$$

Under a displacement transformation $D(\alpha)$ this Hamiltonian reads

$$\begin{aligned}\hat{H}' = &[(-2K\alpha^2\alpha^* + 2\mathcal{E}_\mathrm{p}\alpha^* + \mathcal{E}_0)\hat{a}^\dagger + \mathrm{h.c.}] \\ &+ [(-K\alpha^2 + \mathcal{E}_\mathrm{p})\hat{a}^{\dagger 2} + \mathrm{h.c.}] - 4K|\alpha|^2\hat{a}^\dagger\hat{a} - K\hat{a}^{\dagger 2}\hat{a}^2 - (2K\alpha\hat{a}^{\dagger 2}\hat{a} + \mathrm{h.c.}),\end{aligned} \tag{6}$$

where we have again dropped the constant term $E(\alpha) = -K|\alpha|^4 + \mathcal{E}_\mathrm{p}(\alpha^2 + \alpha^{*2}) + \mathcal{E}_0(\alpha + \alpha^*)$ representing a shift in energy. Following the same steps as above, the coefficient of the $\hat{a}^\dagger$ terms vanish if

$$-2K\alpha^2\alpha^* + 2\mathcal{E}_\mathrm{p}\alpha^* + \mathcal{E}_0 = 0. \tag{7}$$

For small $\mathcal{E}_0$, this equation has three solutions of the form $(\pm\alpha_0 + \epsilon, 0), (\epsilon, 0)$ with $\epsilon = \mathcal{E}_0/4\mathcal{E}_\mathrm{p}$. In practice, we assume that the amplitude of the single-photon drive is small compared to that of the two-photon drive, $\mathcal{E}_0 \ll \mathcal{E}_\mathrm{p}$, so that $\epsilon \to 0$. If the condition Eq. (7) is satisfied, then the Hamiltonian in the displaced frame reduces to

$$\hat{H}' = \left[-\frac{\mathcal{E}_0}{2\alpha^*}\hat{a}^{\dagger 2} + \mathrm{h.c.} - 4K|\alpha|^2\hat{a}^\dagger\hat{a}\right] - K\hat{a}^{\dagger 2}\hat{a}^2 - (2K\alpha\hat{a}^{\dagger 2}\hat{a} + \mathrm{h.c.}). \tag{8}$$

Following Supplementary Note 1, $|0\rangle$ is an eigenstate of $\hat{H}'$ except for the first term which represents an off-resonant parametric drive of strength $|\mathcal{E}_0/2\alpha^*|$ detuned by $4K|\alpha|^2$. If $|\mathcal{E}_0/\alpha| \ll 4K|\alpha|^2$, fluctuations around α are small and $|0\rangle$ remains an eigenstate in the displaced frame. Of the three solutions to Eq. (7), only $(\pm\alpha_0 + \epsilon, 0)$ satisfy the condition $|\mathcal{E}_0/(\pm\alpha_0 + \epsilon)| \ll 4K|\pm\alpha_0 + \epsilon|^2$. The third solution $(\epsilon, 0)$ is unstable because of the large quantum fluctuations around this point. As a result, in the non-displaced frame, the eigenstates of the system are $|\alpha_0 + \epsilon\rangle$ and $|-\alpha_0 + \epsilon\rangle$, where ϵ is a small correction. From the expression for $E(\alpha)$, it also clear that the degeneracy between the eigenstates $|\alpha_0 + \epsilon\rangle$ and $|-\alpha_0 + \epsilon\rangle$ is lifted by an amount $E(\alpha_0) - E(-\alpha_0) = 4\mathcal{E}_0\alpha_0$.

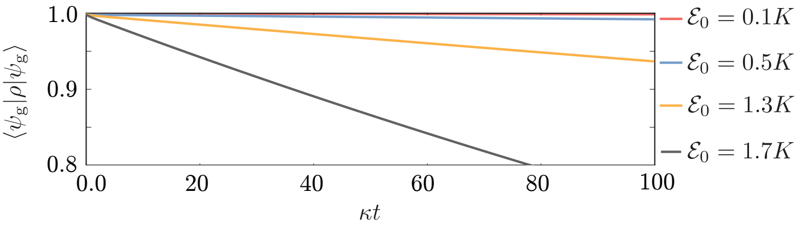

Supplementary Figure 1. **Ground state in presence of single-photon drive**: Time evolution of the probability of a single resonator to remain in the ground state $\langle\psi_g|\hat{\rho}|\psi_g\rangle$, for different single-photon drive strength. The two-photon drive strength is fixed to $\mathcal{E}_\mathrm{p} = 2K$ and $\kappa = 0.2K$.

Increasing $\mathcal{E}_0$, leads to squeezing due to the first term of Eq. (8). As a result, the states are no longer coherent or, in other words, they are no longer the eigenstates of the photon annihilation operator $\hat{a}$ and hence are no longer protected against the single-photon loss channel. To illustrate this effect quantitatively, we numerically diagonalize the Hamiltonian in Eq. (5) to evaluate the ground state for a fixed two-photon drive strength $\mathcal{E}_\mathrm{p} = 2K$ and variable strength single-photon drive amplitude. We numerically solve the master equation for the Hamiltonian in Eq. (5), $\dot{\hat{\rho}} = -i[\hat{H}, \hat{\rho}] + \kappa[\hat{a}\rho\hat{a}^\dagger - (\hat{a}^\dagger\hat{a}\hat{\rho} + \hat{\rho}\hat{a}^\dagger\hat{a})/2]$ with the system initialized to the ground state at $t = 0$ and $\kappa = 0.2K$ [3, 4]. Finally, from these results, we compute the probability of the system to remain in the ground state $\langle\psi_g|\hat{\rho}|\psi_g\rangle$ after time t, presented in Supplementary Figure **1**. As seen from these numerical results, the ground state is invariant to single-photon loss for small $\mathcal{E}_0$, confirming our prediction from the simple theoretical analysis.

SUPPLEMENTARY NOTE 3: EFFECT OF SINGLE PHOTON LOSS DURING THE ANNEALING PROTOCOL FOR A SINGLE DRIVEN KNR

In this section, we provide a simple theoretical analysis to understand the behaviour of the instantaneous ground and excited state during the annealing protocol with a single driven KNR in the presence of single-photon loss. The time-dependent Hamiltonian of this system is given by

$$\hat{H}_1(t) = \left(1 - \frac{t}{\tau}\right)\delta_0\hat{a}^\dagger\hat{a} - K\hat{a}^{\dagger 2}\hat{a} + \left(\frac{t}{\tau}\right)[\mathcal{E}_\mathrm{p}(\hat{a}^{\dagger 2} + \hat{a}^2) + \mathcal{E}_0(\hat{a}^\dagger + \hat{a})]. \tag{9}$$

In the presence of single-photon loss, the dynamics of the system is described by the master equation $\dot{\hat{\rho}} = -i[\hat{H}_{1,\mathrm{eff}}(t)\hat{\rho} - \hat{\rho}\hat{H}^\dagger_{1,\mathrm{eff}}(t)] + \kappa\hat{a}\hat{\rho}\hat{a}^\dagger$, where κ is rate of photon loss and $\hat{H}_{1,\mathrm{eff}}(t) = \hat{H}_1(t) - \kappa\hat{a}^\dagger\hat{a}/2$. Following Supplementary Notes 1 and 2, we apply a displacement transformation $D(\alpha)$ to $\hat{H}_{1,\mathrm{eff}}(t)$

$$\begin{aligned}\hat{H}'_{1,\mathrm{eff}} &= [(-2K\alpha^2\alpha^* + 2\mathcal{E}_\mathrm{p}(t)\alpha^* + \delta(t)\alpha - i\frac{\kappa}{2}\alpha + \mathcal{E}_0(t))\hat{a}^\dagger + \mathrm{h.c.}] \\ &\quad + [(-K\alpha^2 + \mathcal{E}_\mathrm{p})\hat{a}^{\dagger 2} + \mathrm{h.c.}] - 4K|\alpha|^2\hat{a}^\dagger\hat{a} + \delta(t)\hat{a}^\dagger\hat{a} - i\frac{\kappa}{2}\hat{a}^\dagger\hat{a} - K\hat{a}^{\dagger 2}\hat{a}^2 - (2K\alpha\hat{a}^{\dagger 2}\hat{a} + \mathrm{h.c.}).\end{aligned} \tag{10}$$

For convenience, the variables have been redefined as $\delta(t) = \delta_0(1 - t/\tau)$, $\mathcal{E}_\mathrm{p}(t) = \mathcal{E}_\mathrm{p}t/\tau$ and $\mathcal{E}_0(t) = \mathcal{E}_0 t/\tau$. Again, we take α to satisfy

$$-2K\alpha^2\alpha^* + 2\mathcal{E}_\mathrm{p}(t)\alpha^* + \delta(t)\alpha - i\frac{\kappa}{2}\alpha + \mathcal{E}_0(t) = 0, \tag{11}$$

such that the effective Hamiltonian reads

$$\hat{H}'_{1,\mathrm{eff}} = [(-K\alpha^2 + \mathcal{E}_\mathrm{p})\hat{a}^{\dagger 2} + \mathrm{h.c.}] - 4K|\alpha|^2\hat{a}^\dagger\hat{a} + \delta(t)\hat{a}^\dagger\hat{a} - i\frac{\kappa}{2}\hat{a}^\dagger\hat{a} - K\hat{a}^{\dagger 2}\hat{a}^2 - (2K\alpha\hat{a}^{\dagger 2}\hat{a} + \mathrm{h.c.}). \tag{12}$$

Eq. (11) admits three solutions: $\sim (0, 0), (\pm\alpha'_0, 0)$, with $\alpha'_0 = \sqrt{[2\mathcal{E}_\mathrm{p}(t) + \delta(t)]/2K}$. Repeating the procedure described in Supplementary Note 1 and 2 we find, at short times when $\delta(t) \gg 2\mathcal{E}_\mathrm{p}(t)$, that $|0\rangle$ is approximately the lower energy, stable eigenstate of the Hamiltonian. As the evolution proceeds, the strength of the detuning and two-photon drive is

modified as $\delta(t) \ll 2\mathcal{E}_{\rm p}(t)$. In this case $(0,0)$ is not a stable eigenstate. On the other hand, at $(\pm\alpha_0', 0)$ the Hamiltonian reads

$$\hat{H}_{1,\rm eff}' = \frac{1}{2}\left[\left\{-\left(\delta(t) - i\frac{\kappa}{2}\right)\frac{\alpha}{2\alpha_0'^*} - \frac{\mathcal{E}_0}{\alpha_0'^*}\right\}\hat{a}^{\dagger 2} + \text{h.c.}\right] - 4K|\alpha_0'|^2\hat{a}^\dagger\hat{a} + \delta(t)\hat{a}^\dagger\hat{a} - i\frac{\kappa}{2}\hat{a}^\dagger\hat{a} - K\hat{a}^{\dagger 2}\hat{a}^2 - (2K\alpha_0'\hat{a}^{\dagger 2}\hat{a} + \text{h.c.}). \tag{13}$$

In the absence of the first term, $|0\rangle$ would be the eigenstate of the above Hamiltonian and hence the coherent states $|\pm\alpha_0'\rangle$ the eigenstates in the non-displaced frame. The first term represents a parametric drive of amplitude $\zeta = |-(\delta(t) - i\kappa/2)(\alpha_0'/2\alpha_0'^*) - \mathcal{E}_0/\alpha_0'^*|$ and detuned by $|-4K|\alpha_0'|^2\hat{a}^\dagger\hat{a} + \delta(t)\hat{a}^\dagger\hat{a} - i\kappa/2|$. In other words, the effect of detuning, photon-loss and single-photon drive is to squeeze the fluctuations around $(\pm\alpha_0', 0)$. When $\delta(t) < 2\mathcal{E}_{\rm p}(t)$, $\kappa < 8\mathcal{E}_{\rm p}(t)$ and $\mathcal{E}(t) < 4K|\alpha_0'|^3$ then squeezing is negligible and the eigenstates in the non-displaced frame are approximately coherent states $|\pm\alpha_0'\rangle$. If $\mathcal{E}_0 > 0$, then $(-\alpha_0', 0)$ corresponds to the lower energy state, and, on the other hand, if $\mathcal{E}_0 < 0$, $(\alpha_0', 0)$ corresponds to the lower energy state.

Importantly, the amplitude of the squeezing drive for the lower energy state, $|\zeta| = |-[\delta(t) - |\mathcal{E}(t)|/\sqrt{2\mathcal{E}_{\rm p}(t) + \delta(t)/2K}] - i\kappa/2|$, is smaller than that for higher energy state, $|\zeta| = |-[\delta(t) + |\mathcal{E}(t)|/\sqrt{2\mathcal{E}_{\rm p}(t) + \delta(t)/2K}] - i\kappa/2|$. As a result, the deviation of the lower energy state from a coherent state is smaller than the similar deviation of the higher energy state. For this reason, during the adiabatic evolution, the lower energy state which is the computational ground state is more stable against photon-jump operation. On the other hand, with $\delta(t)$ having the opposite sign, $|\zeta|$ for the higher energy state becomes smaller than that for the lower energy state. As a result, the instantaneous excited state is then more stable than the instantaneous ground state. This further exemplifies that stability is related to the nature of quantum fluctuations rather than to energy. In the main body of the paper, we take $\delta(t) > 0$ to ensure that the instantaneous ground state is more stable. One could just as easily have chosen $\delta(t) < 0$ and inverted the Ising problem so that the desired solution would be given by the excited state which would then be more stable to single photon loss.

SUPPLEMENTARY NOTE 4: EFFECT OF FAST-ROTATING TERMS

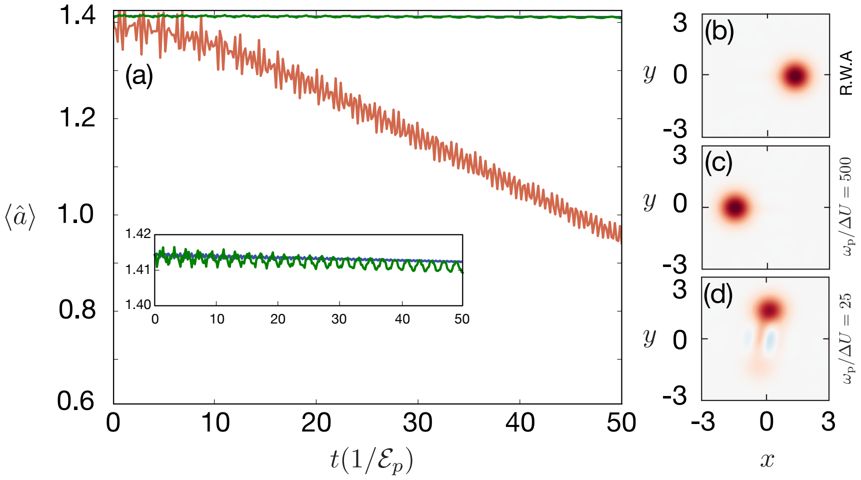

Supplementary Figure 2. Amplitude of field with and without RWA: (a) Time dependence of the field $|\langle\hat{a}\rangle|$ in a two-photon driven KNR initialized to $|\alpha_0\rangle$ in the presence of single-photon loss and without making the rotating wave approximation (RWA). By increasing the ratio of frequency of the two-photon drive ω_p to the tunneling barrier $\Delta U = \mathcal{E}_{\rm p}^2/K$, the probability of resonant excitations exponentially decreases and the field amplitude remains essentially constant at its original value. The red and green lines correspond to $\omega_{\rm p}/\Delta U = 25$ and 500, respectively. The blue line is obtained under the RWA. The inset shows an enlarged view of the non-RWA results with $\omega_{\rm p}/\Delta U = 500$ (green line) and RWA results (blue line). The other parameters are $\kappa = K/100$, $\mathcal{E}_{\rm p} = 2K$, $\alpha_0 = \sqrt{2}$ and $\Delta U = 4K$. Wigner function (b) in the rotating frame at time $t = 50/\mathcal{E}_{\rm p}$ under RWA, (c) in the laboratory frame without RWA and $\omega_{\rm p}/\Delta U = 500$, and (d) in the laboratory frame without RWA and $\omega_{\rm p}/\Delta U = 25$.

The two-photon driven KRN Hamiltonian of Eq. (1) describes the physics of the system in a frame rotating at the 2-photon drive frequency. To obtain this Hamiltonian, fast rotating terms have been dropped following the standard

rotating wave approximation (RWA). As discussed in Supplementary Note 1, under that approximation the coherent states $|\pm\alpha_0\rangle$ are eigenstates of the system.

The effect of the conventionally disregarded fast rotating terms has been studied in Ref. [5] where it was shown that these terms can lead to tunneling between $|\alpha_0\rangle$ and $|-\alpha_0\rangle$. This tunneling is a result of resonant transitions between $|\pm\alpha_0\rangle$ and other eigenstates of the two-photon driven KNR. Fortunately for the present situation, the corresponding transition rate decreases exponentially with an increase of the ratio of frequency of the two-photon drive ω_p and the tunneling barrier which, in the notation of Supplementary Note 1, is $\Delta U = \mathcal{E}_\mathrm{p}^2/K$ [5]. This is because of increasing difference between the momentum of the states $|\pm\alpha_0\rangle$ and the other eigenstates which can resonantly mix with $|\pm\alpha_0\rangle$ also increases with ω_p (see Eq. (12) and Fig. (3) in Ref. [5]). Given the parameters that we suggest using, and which are realizable in the laboratory, this tunneling is in practice negligible.

This can be illustrated with a numerical example. For this purpose, consider the Hamiltonian of the two-photon driven KNR without the RWA which reads

$$\hat{H}(t) = \omega_\mathrm{r}\hat{a}^\dagger\hat{a} - K\hat{a}^{\dagger 2}\hat{a}^2 + 2\mathcal{E}_\mathrm{p}\cos(\omega_\mathrm{p}t)(\hat{a}^{\dagger 2} + \hat{a}^2), \tag{14}$$

with $\omega_\mathrm{p} = 2\omega_\mathrm{r}$. In the presence of single-photon loss, the dynamics of the system is described by the master equation

$$\dot{\hat{\rho}} = -i[\hat{H}(t), \hat{\rho}] + \kappa\left(\hat{a}\hat{\rho}\hat{a}^\dagger - \frac{1}{2}\hat{a}^\dagger\hat{a}\hat{\rho} - \frac{1}{2}\hat{\rho}\hat{a}^\dagger\hat{a}\right). \tag{15}$$

Before considering the effect of the non-RWA terms, it is worth pointing out that the presence of damping leads to a transfer of population between the non-orthogonal states $|\pm\alpha_0\rangle$ at a rate $\kappa\alpha_0^2\exp(-2|\alpha_0|^2)$. This effect if negligibly small at large enough α_0 and for the small values of κ that are relevant here.

In the presence of the non-RWA terms, the states $|\alpha_0\rangle$ are no longer the exact eigenstates of the system. In this situation, the tunneling rate increases due to mixing with other eigenstates. This is illustrated in Fig. **2**a) which shows $\langle\hat{a}\rangle$ as a function of time obtained numerically by solving Eq. (15) with $\omega_\mathrm{p}/\Delta U = 25$ (full red line) and 500 (full green line) for $\alpha_0 = \sqrt{2}$. The dashed blue line on this Figure shows the equivalent result under the RWA. The transition between the two coherent states is readily apparent for the small value of $\omega_\mathrm{p}/\Delta U = 25$. On the other hand, apart from rapid oscillations (see inset), the more relevant case $\omega_\mathrm{p}/\Delta U = 500$ is barely distinguishable from the RWA results.

Fig. **2**d) shows the numerically evaluated Wigner function in the lab frame at time $t = 0$ and $t = 50/\mathcal{E}_\mathrm{p}$. The appearance of the additional peak and interference fringe pattern at $t = 50/\mathcal{E}_\mathrm{p}$ signifies the enhanced tunneling due to the fast-rotating terms when $\omega_\mathrm{p}/\Delta U = 25$. On the other hand, when $\omega_\mathrm{p}/\Delta U = 500$, the fringe pattern and the additional peak is diminished, as illustrated by the Wigner function in Fig. **2**c). In a realistic implementation of this scheme ω_p is typically of the order of a $10-20$ GHz, whereas, ΔU is of the order of 10-20 MHz, which means that $\omega_\mathrm{p}/\Delta U > 500$ and the tunneling due to resonant excitations via the fast rotating terms will not adversely affect the annealing schedule. Note that the rotations in the Wigner function in Fig. **2**c,d) is due to the choice of working in the lab frame. The rotation in the coherent state is not seen if we instead work in the rotating frame as is shown in Fig. **2**b).

In addition to non-RWA corrections, the Hamiltonian of realistic superconducting circuit implementations will have higher order non-linearities than Eq. (14). To verify that these higher-order terms do not change the above conclusion, we consider the time-dependent annealing Hamiltonian for the problem of a single-spin in magnetic field with the full cosine potential of the Josephson junction,

$$\hat{H}_1(t) = \omega_\mathrm{r}\hat{a}^\dagger\hat{a} + i\mathcal{E}(t)(\hat{a}^\dagger - \hat{a}) - 2\left\{E_\mathrm{J}\cos\left(\frac{\Phi(t)}{\phi_0}\right)\cos\left(\frac{\hat{\phi}}{\phi_0}\right) + \frac{1}{2}\frac{\hat{\phi}^2}{\phi_0^2}\right\} \tag{16}$$

In the above expression, $\phi_0 = \hbar/2e$ is the flux quanta, $\hat{\phi} = \phi(\hat{a}^\dagger + \hat{a})$ where ϕ the zero-point fluctuations of the resonator mode and E_J the Josephson energy. Recall the annealing protocol, in which the external flux $\Phi(t)$ is modulated close to twice the frequency of the resonator to obtain the two-photon drive and its amplitude is adiabatically increased. In the numerical simulations, we take $\Phi(t) = \Phi_x\phi_0 + \delta\Phi_x(t/\tau)\phi_0\cos[2\omega_\mathrm{r}t + \delta_0 t(1 - t/2\tau)]$ with τ the final time and δ_0 is the initial detuning (as defined in the manuscript). Similarly, the single-photon drive is modulated as $\mathcal{E}(t) = 2\mathcal{E}_0(t/\tau)\sin(\omega_\mathrm{r}t + \delta_0 t(1 - t/2\tau)/2)$. Here $\mathcal{E}_0$ is the single photon drive required to encode the problem of a single spin in the magnetic field, as described in the manuscript. With these values, the effective two-photon drive and Kerr nonlinearity are $\mathcal{E}_\mathrm{p} = 2E_\mathrm{J}\phi^2\sin(\Phi_x)\sin(\delta\Phi_x)/4$, $K = 2E_\mathrm{J}\phi^4\cos(\Phi_x)\cos(\delta\Phi_x)/4$ and we take $\Phi_x = 0.2$ and $\delta\Phi_x = 0.03$. The resonator is initialized to vacuum at $t = 0$ and its state at $t = \tau$ is determined by evolving under the Hamiltonian $\hat{H}_1(t)$. The parameters used in the simulation are such that the effective barrier potential $\Delta U = 14.45K$, $\mathcal{E}_\mathrm{p} \sim 1.95K$, $\omega_\mathrm{r} \sim 4984.4K$, $\mathcal{E}_0 = 0.8K$, $\delta_0 = 0.8K$ and $\tau = 250/K$. We numerically simulate the evolution of the state of the resonator under the annealing Hamiltonian in Eq. (16) and Fig. **3** illustrate the resulting Wigner function in the laboratory frame at $t = \tau$ for (a) $\mathcal{E}_0 > 0$ and (b) $\mathcal{E}_0 < 0$. For the reasonable choice of parameters made here, the final

state is essentially not affected by higher-order nonlinearities or fast rotating terms. Indeed, apart from a rotation due to the choice of frame, the result is very close to coherent states and follows the expected dependence of the sign of the single-photon drive. For $\mathcal{E}_0 > 0$ and $\mathcal{E}_0 < 0$ the overlap of the final state of the resonator with the coherent states $|\pm\alpha\rangle$ where $\alpha = 1.37 + 1.44i$ is 99.92%. This large overlap with coherent states indicates that for $\omega_{\rm p}/\Delta U \sim 690$ the effect of fast rotating terms is small and do not cause tunneling to the incorrect state, thereby ensuring that a high success probability of 99.92% is obtained.

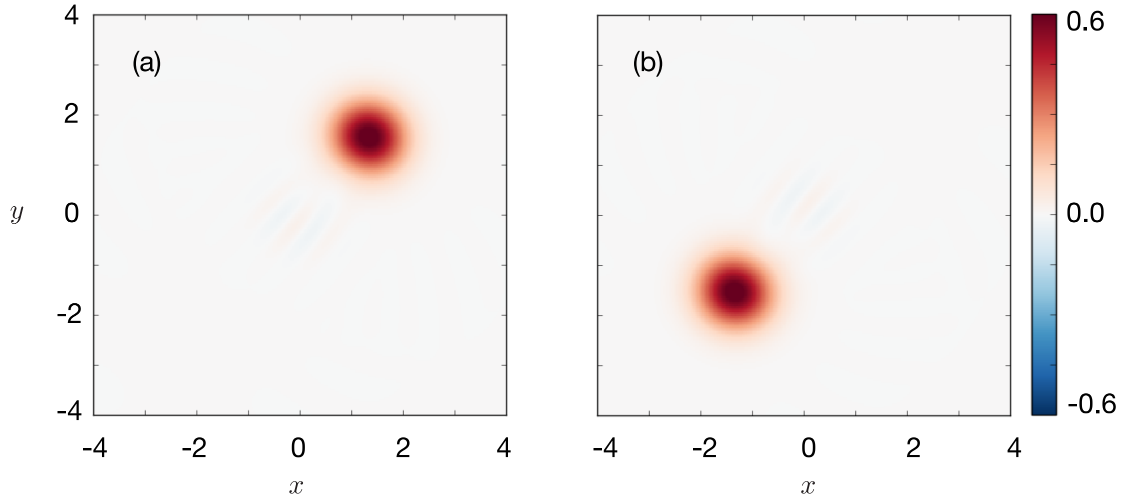

Supplementary Figure 3. **Wigner function in the laboratory frame after the annealing schedule:** (a) single-photon drive amplitude $\mathcal{E}_0 > 0$ and (b) $\mathcal{E}_0 < 0$. Evolution is simulated using the full Cosine potential given of Eq. (16). As expected, the final coherent states for $\mathcal{E}_0 > 0$ and $\mathcal{E}_{<}0$ are separated by a phase of π.

SUPPLEMENTARY NOTE 5: ENERGY SPECTRUM AND AVERAGE SUCCESS PROBABILITY OF PROBLEMS ENCODED ON A SINGLE PLAQUETTE

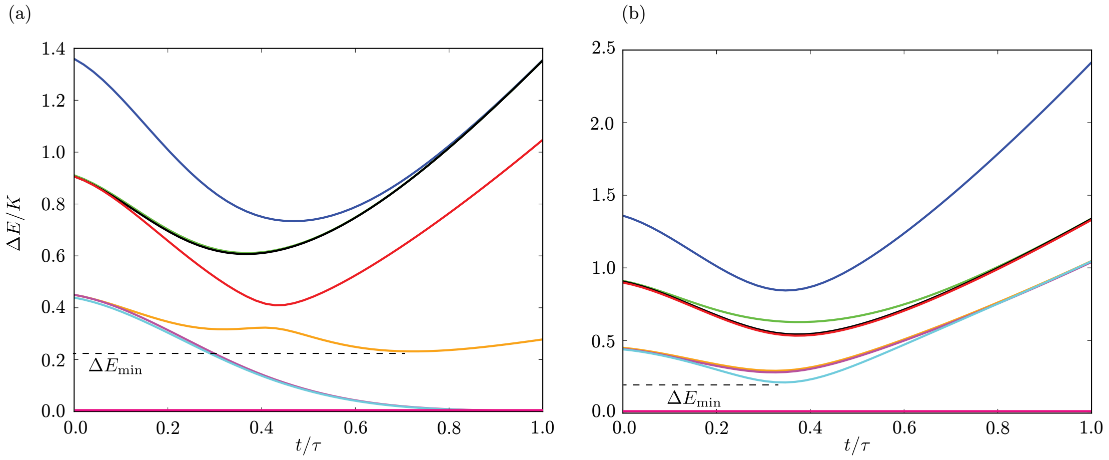

Supplementary Figure 4. **Evolution of the energy spectrum during the adiabatic evolution:** The figure shows the time-dependent energy spectrum during the adiabatic protocol for the fully connected three spin problem on a plaquette when the interactions between the spins is (a) anti-ferromagnetic, in which case the ground state of the problem in the physical spin basis is three fold-degenerate and (b) ferromagnetic, in which case the ground state of the problem in the physical spin basis is non-degenerate. The energy is measured with respect to the ground state $\Delta E = E_i - E_0$.

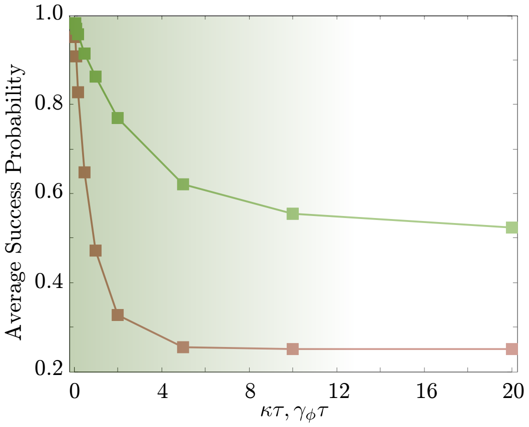

Supplementary Figure 5. **Average success probability for all problem instances on a plaquette:** The figure shows the dependence of the average success probability for all fully connected problems on a single plaquette when the adiabatic protocol is implemented using KNRs (green squares) characterized by single photon loss rate κ and qubits (red squares) characterized by the dephasing rate γ_ϕ. The total computation time τ is the same for both cases.

There are eight fully-connected Ising problems for three logical spins with weights $J_{i,j} = \pm 1$: one where all spins are ferromagnetically coupled, one where all are anti-ferromagnetically coupled, three problems where two spins are ferromagnetically coupled, while the coupling to the third is anti-ferromagnetic and three problems where two spins are anti-ferromagnetically coupled, while the coupling to the third is ferromagnetic. All these problems can be encoded on a single plaquette by appropriately choosing the sign of the single-photon drive. For example, as shown in the manuscript, the problem with anti-ferromagnetic coupling between all logical spins is embedded on the KNRs by applying single-photon drives such that $J > 0$. Similarly, to embed a problem where all logical spins are coupled ferromagnetically, all the applied single-photon drives must be such that $J < 0$. In order to embed the problem correctly in the LHZ scheme, the four-body coupling must be larger than local magnetic fields [6]. In our case, this implies $C|\alpha_0|^3 > J$.

To illustrate a particular case, we consider all the logical spins to be coupled anti-ferromagnetically. The ground state is then sixfold degenerate: $\{|0,0,1\rangle, |0,1,0\rangle, |1,0,0\rangle, |1,1,0\rangle, |1,0,1\rangle, |0,1,1\rangle\}$. In the basis of physical spins, which map the relative configuration of the logical spins, the ground state is threefold degenerate: $\{|\bar{0},\bar{0},\bar{1}\rangle, |\bar{0},\bar{1},\bar{0}\rangle, |\bar{1},\bar{0},\bar{0}\rangle\}$. Supplementary Figure **4**(a) shows the change in the energy spectrum during the adiabatic protocol with $\mathcal{E}_\mathrm{p} = 2K$, $C = 0.05K$ and single-photon drive to all the resonators $J = 0.095K$. The energy is referred to the ground state. As expected, at $t = \tau$, the ground state becomes threefold degenerate.

On the other hand, if the logical spins are coupled ferromagnetically, then there are two possible ground states: $\{|0,0,0\rangle, |1,1,1\rangle\}$. This implies that, in the physical spin basis, there is a single non-degenerate ground state: $|\bar{1},\bar{1},\bar{1}\rangle$. Supplementary Figure **4**(b) shows the change in the energy spectrum during the adiabatic protocol with $\mathcal{E}_\mathrm{p} = 2K$ and $C = 0.05K$ but with the single-photon drive $J = -0.095K$ on all the resonators.

In a similar way, it is possible to encode all eight problems on the plaquette. We find that, in the absence of single-photon loss, the average success probability to find the correct ground state with the adiabatic algorithm is 99.7% in time $\tau = 200/K$. Supplementary figure **5** shows the dependence of the success probability on the rate of single-photon loss. It also presents the success probability of the protocol implemented using qubits with dephasing rate γ_ϕ. The time-dependent Hamiltonian for qubits is designed to have the same minimum energy gap as that with the JPAs and the duration of the protocol is also chosen to be the same. Clearly the adiabatic protocol with the JPAs outperform that with qubits in the presence of equal strength noise.

SUPPLEMENTARY NOTE 6: PHYSICAL REALIZATION OF A PLAQUETTE

Josephson parametric amplifiers (JPAs) are arranged in a triangular lattice and coupled together using Josephson junctions (JJ). As described in the main text, a single plaquette is comprised of four JPAs and a coupling JJ. The JPA is realized by embedding a SQUID in a resonator and modulating the flux through the SQUID at a frequency close to twice the frequency of the resonator [1, 7–9]. To implement the adiabatic protocol described in the manuscript (see Eq. (2) and Methods section in the manuscript), the flux modulation frequency is linearly varied from $\omega_{\mathrm{p},k}(0) = 2\omega_{\mathrm{r},k} - 2\delta_0$ at $t = 0$ to $\omega_{\mathrm{p},k}(\tau) = 2\omega_{\mathrm{r},k} - \delta_0$ at $t = \tau$. With an additional single-photon drive, the Hamiltonian of the kth JPA can

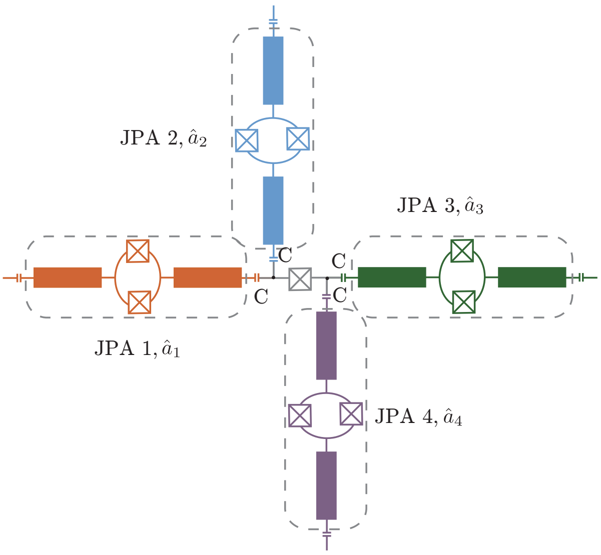

Supplementary Figure 6. **Illustration of the circuit for physical realization of the plaquette:** Four JPAs are linearly coupled to a mode of a Josephson junction via capacitors. The JPA modes are far detuned from the junction mode so that the coupling between them is dispersive. The non-linearity of the junction induces a four-body coupling between the JPAs if the frequencies of the two-photon drives to the JPAs are such that $\omega_{\mathrm{p},1} + \omega_{\mathrm{p},2} = \omega_{\mathrm{p},3} + \omega_{\mathrm{p},4}$.

then be written as

$$\hat{H}_{\mathrm{JPA},k} = \omega_{\mathrm{r},k}\hat{a}_k^\dagger \hat{a}_k - K\hat{a}_k^{\dagger 2}\hat{a}_k^2 + J(t)[e^{-i\omega_{\mathrm{p},k}(t)t/2}\hat{a}^\dagger + e^{i\omega_{\mathrm{p},k}(t)t/2}\hat{a}] + \mathcal{E}_{\mathrm{p}}(t)[e^{-i\omega_{\mathrm{p},k}(t)t}\hat{a}^{\dagger 2} + e^{i\omega_{\mathrm{p},k}(t)t}\hat{a}^2]. \tag{17}$$

The Kerr nonlinearity K and frequency $\omega_{\mathrm{r},k}$ are determined by the charging and Josephson energy of the junctions in the SQUID. The two-photon drive $\mathcal{E}_{\mathrm{p}}$ depends on the magnitude of the flux modulation.

Consider Supplementary Figure **6** which shows four JPAs of different frequencies (indicated by different colors) capacitively coupled to a single JJ. The Hamiltonian of this plaquette is given by

$$\hat{H} = \sum_{k=1}^{4} \hat{H}_{\mathrm{JPA},k} + \hat{H}_{\mathrm{c}}, \tag{18}$$

where the Hamiltonian of each JPA is given in Eq. (17) and the coupling Hamiltonian is given by

$$\begin{aligned}\hat{H}_{\mathrm{c}} = {} & \omega_c \hat{a}_c^\dagger \hat{a}_c + g_1(\hat{a}_c^\dagger \hat{a}_1 + \hat{a}_1^\dagger \hat{a}_c) + g_2(\hat{a}_c^\dagger \hat{a}_2 + \hat{a}_2^\dagger \hat{a}_c) - g_3(\hat{a}_c^\dagger \hat{a}_3 + \hat{a}_3^\dagger \hat{a}_c) - g_4(\hat{a}_c^\dagger \hat{a}_4 + \hat{a}_4^\dagger \hat{a}_c) \\ & - E_J\left(\cos\left(\frac{\hat{\Phi}}{\phi_0}\right) + \frac{1}{2}\left(\frac{\hat{\Phi}}{\phi_0}\right)^2\right),\end{aligned} \tag{19}$$

where $\hat{\Phi} = \phi_c(\hat{a}_c^\dagger + \hat{a}_c)$, $\phi_0 = \hbar/2e$ is the flux quantum, $\hat{a}_c$ $(\hat{a}_c^\dagger)$ is the annihilation (creation) operator for the mode across the coupling JJ and ϕ_c is the standard deviation of the zero-point flux fluctuation for this JJ mode. $\hat{\Phi}$ is the phase across the junction, E_J is its Josephson energy, ω_c is the frequency of the junction mode and g_k is the rate at which energy is exchanged between this mode and the kth JPA. Following the definition for the mode operators, the quadratic term $\propto \hat{\Phi}^2$ has been removed from the cosine. The coupling rate can be expressed as $g_k = \phi_0^2 e^2/2C\phi_c\phi_k$ in terms of the coupling capacitor C and the zero point flux fluctuation for the JPA mode (ϕ_k). The total Hamiltonian is obtained by substituting Eq. (19) and Eq. (17) in Eq. (18).

The frequency of the junction mode is designed to be largely detuned from the JPA modes, such that $\Delta_{\mathrm{k}} = \omega_c - \omega_{\mathrm{r},k} \gg g_k$. It is then possible to apply a dispersive unitary transformation $\hat{U} = \exp(-i(g_1/\Delta_1)\hat{a}_c^\dagger \hat{a}_1 - i(g_2/\Delta_2)\hat{a}_c^\dagger \hat{a}_2 + i(g_3/\Delta_3)\hat{a}_c^\dagger \hat{a}_3 + i(g_4/\Delta_4)\hat{a}_c^\dagger \hat{a}_4 + \mathrm{h.c.})$ to the total Hamiltonian, so that, to the second order in g_k/Δ_k, the total

Hamiltonian becomes

$$\hat{H} \sim \sum_{k=1}^{4}\left(\hat{H}_{\mathrm{JPA},k} - \frac{g_k^2}{\Delta_k}\hat{a}_k^\dagger\hat{a}_k\right) + \left(\omega_c + \frac{g_k^2}{\Delta_k}\right)\hat{a}_c^\dagger\hat{a}_c - E_J\left(\cos\left(\frac{\hat{\Phi}'}{\phi_0}\right) + \frac{1}{2}\left(\frac{\hat{\Phi}'}{\phi_0}\right)^2\right), \tag{20}$$

$$\sim \sum_{k=1}^{4}\left(\hat{H}_{\mathrm{JPA},k} - \frac{g_k^2}{\Delta_k}\hat{a}_k^\dagger\hat{a}_k\right) + \left(\omega_c + \frac{g_k^2}{\Delta_k}\right)\hat{a}_c^\dagger\hat{a}_c - E_J\frac{1}{4!}\frac{\hat{\Phi}'^4}{\phi_0^4}, \tag{21}$$

where

$$\hat{\Phi}' = \phi_c\left(\hat{a}_c^\dagger - \frac{g_1}{\Delta_1}\hat{a}_1^\dagger - \frac{g_2}{\Delta_2}\hat{a}_2^\dagger + \frac{g_3}{\Delta_3}\hat{a}_3^\dagger + \frac{g_4}{\Delta_4}\hat{a}_4^\dagger + \mathrm{h.c.}\right). \tag{22}$$

The expression in Eq. (21) is obtained by expanding the cosine term to fourth order.

The central Josephson junction mode is far detuned from the JPAs and is not driven externally so that we have $\langle\hat{a}_c\rangle = \langle\hat{a}_c^\dagger\hat{a}_c\rangle = 0$. As a result, it is possible to eliminate this mode and obtain a Hamiltonian for the JPA modes only. Furthermore, in a frame rotating at the frequencies of the two-photon drives, the photon annihilation operators transform as $\hat{a}_k \to e^{-i\omega_{\mathrm{p},k}(t)t}\hat{a}_k$. As discussed in the text, the two-photon drive frequencies are such that $\omega_{\mathrm{p},k} \neq \omega_{\mathrm{p},m}$ and $\omega_{\mathrm{p},1} + \omega_{\mathrm{p},2} = \omega_{\mathrm{p},3} + \omega_{\mathrm{p},4}$. As a result, we can eliminate fast rotating terms in the expansion of the last term in Eq. (21) and realize the plaquette Hamiltonian

$$\hat{H}_{\mathrm{plaquette}} \sim \sum_{k=1}^{4}\left(\hat{H}_{\mathrm{JPA},k} - \frac{g_k^2}{\Delta_k}\hat{a}_k^\dagger\hat{a}_k\right) - E_J\frac{\phi_c^4}{\phi_0^4}\frac{g_1g_2g_3g_4}{\Delta_1\Delta_2\Delta_3\Delta_4}(\hat{a}_1^\dagger\hat{a}_2^\dagger\hat{a}_3\hat{a}_4 + \mathrm{h.c.}) - E_J\frac{\phi_c^4}{\phi_0^4}\sum_{k\neq m=1}^{4}\frac{g_k^2g_m^2}{\Delta_k^2\Delta_m^2}\hat{a}_k^\dagger\hat{a}_k\hat{a}_m^\dagger\hat{a}_m. \tag{23}$$

The second part of the first term results in a frequency shift of the JPA modes due to off-resonant coupling with the JJ and only leads to a renormalization of the energies. The second term in the above expression is the desired four-body coupling between the JPAs. The four-body coupling strength C defined in the manuscript can be written in terms of circuit parameters as $C = E_J\frac{\phi_c^4}{\phi_0^4}\frac{g_1g_2g_3g_4}{\Delta_1\Delta_2\Delta_3\Delta_4}$. As an example, choosing $E_J/2\pi = 600$ GHz, $\phi_c = 0.12\phi_0$, $g_k/\Delta_k \sim 0.12$ we estimate $C/2\pi = 63$ KHz. For a typical strength of Kerr nonlinearity $K/2\pi = 600$ KHz this leads to $C/K \sim 0.1$. The last term gives rise to a cross-Kerr interaction between the JPAs. As discussed in the following section, if the amplitude of the coherent states forming the computational subspace is large, then this term does not affect the structure of the energy spectrum.

SUPPLEMENTARY NOTE 7: EFFECT OF THE CROSS-KERR COUPLING BETWEEN THE JPAS

In the computational subspace, the cross-Kerr coupling between the JPAs can be written as, $\hat{a}_k^\dagger\hat{a}_k\hat{a}_m^\dagger\hat{a}_m = |\alpha_0|^4e^{-4|\alpha_0|^2}\hat{\sigma}_{k,\mathrm{x}}\otimes\hat{\sigma}_{m,\mathrm{x}} + \mathrm{const.}$, where $\sigma_{k,\mathrm{x}}$ is the Pauli operator $|\bar{1}\rangle\langle\bar{0}| + |\bar{0}\rangle\langle\bar{1}|$. If α_0 is large then $e^{-4|\alpha_0|^2} \to 0$ and the cross-Kerr term only leads to a constant shift in energy without inducing any errors in the encoding. This can be confirmed by numerically evaluating the energy spectrum, for example, for the frustrated three spin problem on a single plaquette. For $C|\alpha_0|^3 > J$, we expect that, at the end of the adiabatic protocol, the ground state is triple-fold degenerate $\{|\bar{0},\bar{0},\bar{1}\rangle, |\bar{0},\bar{1},\bar{0}\rangle, |\bar{1},\bar{0},\bar{0}\rangle\}$, where $|\bar{0}/\bar{1}\rangle = |\pm\alpha_0\rangle$. However, in presence of the cross-Kerr terms, we find that that the degeneracy is lifted by $\sim 0.005K$ if $\alpha_0 = \sqrt{2}$. If, on the other hand, $\alpha_0 = \sqrt{3}$, then the lift in the degeneracy reduces to $0.0004K$. This confirms that as α_0 increases, the errors due to the cross-Kerr terms decrease.

Furthermore, to ensure that the cross-Kerr terms do not induce additional dephasing in the presence of single-photon loss, we repeat the numerical simulations for the problem of three coupled spins embedded on a plaquette with these terms included and determine the average success probability. The numerical simulations are limited by the size of the Hilbert space and we take $\alpha_0 = \sqrt{2}$ with the time-dependent Hamiltonian

$$\hat{H} = \left(1 - \frac{t}{\tau}\right)\hat{H}_i + \left(\frac{t}{\tau}\right)\hat{H}_p, \tag{24}$$

where

$$\begin{aligned}
\hat{H}_i &= \sum_{k=1}^{3}(\delta_0\hat{a}_k^\dagger\hat{a}_k - K\hat{a}_k^{\dagger2}\hat{a}_k^2) - (C\hat{a}_1^\dagger\hat{a}_2^\dagger\hat{a}_3\hat{a}_4 + \mathrm{h.c.}) - C\sum_{k,m=1}^{3}\hat{a}_k^\dagger\hat{a}_k\hat{a}_m^\dagger\hat{a}_m,\\
\hat{H}_p^{\mathrm{LHZ}} &= \sum_{k=1}^{3}\{(1-f)\delta_0\hat{a}_k^\dagger\hat{a}_k - K\hat{a}_k^{\dagger2}\hat{a}_k^2 + \mathcal{E}_{\mathrm{p}}(\hat{a}_k^{\dagger2} + \hat{a}_k^2) + J(\hat{a}_k^\dagger + \hat{a}_k)\} - (C\hat{a}_1^\dagger\hat{a}_2^\dagger\hat{a}_3\hat{a}_4 + \mathrm{h.c.}) - C\sum_{k,m=1}^{3}\hat{a}_k^\dagger\hat{a}_k\hat{a}_m^\dagger\hat{a}_m,\\
\hat{H}_{\mathrm{fixed}} &= -K\hat{a}_4^{\dagger2}\hat{a}_4^2 + \mathcal{E}_{\mathrm{p}}(\hat{a}_4^{\dagger2} + \hat{a}_4^2).
\end{aligned} \tag{25}$$

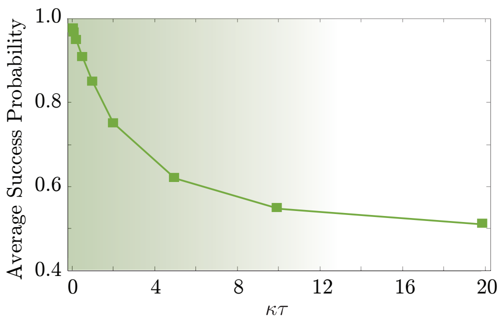

Supplementary Figure 7. **Average success probability for all problem instances on a plaquette with residual interactions:** The figure shows the dependence of the average success probability for all fully connected problems on a single plaquette with the $\sum_{k,m=1}^{3} \hat{a}_k^\dagger \hat{a}_k \hat{a}_m^\dagger \hat{a}_m$ term included.

In contrast to Eq. (2) in the main text, we have included here the cross-Kerr terms. In addition, we have included additional detuning to the problem Hamiltonian $\propto (1-f)$ in order to remove the above mentioned lifting of degeneracy for small α_0. In the example presented here we use $f = 0.7$. The average success probability for all the eight possible problems on the plaquette, shown in Fig. **7**, effectively does not change compared to the case when the $\sum_{k,m=1}^{3} \hat{a}_k^\dagger \hat{a}_k \hat{a}_m^\dagger \hat{a}_m$ term is neglected.

SUPPLEMENTARY NOTE 8: TUNABLE FOUR-BODY COUPLING WITH A JOSEPHSON RING MODULATOR

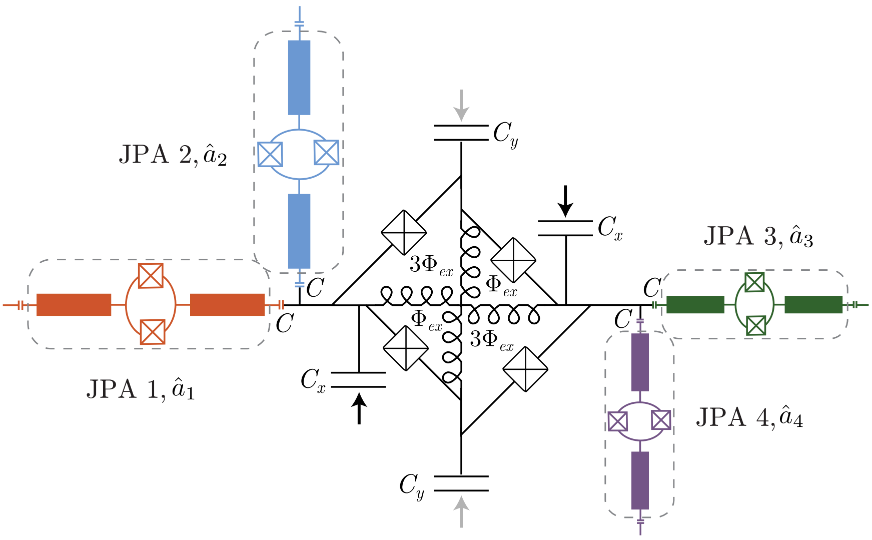

Supplementary Figure 8. **Tunable four-body coupling with JRM:** The figure illustrates the circuit for implementing a tunable four-body interaction using a Josephson ring modulator (JRM). Classical microwave drives of equal strength but opposite phase, as shown with the dark and light arrows, activates the four-body coupling between the JPAs.

An alternative way to implement a four-body interaction in a tunable way is by using an imbalanced shunted Josephson Ring Modulator (JRM) as illustrated in Supplementary Figure **8**. The JRM consists of 3 orthogonal mutually interacting modes $\hat{\phi}_x, \hat{\phi}_y$ and $\hat{\phi}_z$ [10, 11], which also couple with the JPA modes. The Hamiltonian can be

expressed as

$$\hat{H}_c = \omega_x \hat{a}_x^\dagger \hat{a}_x + \omega_y \hat{a}_y^\dagger \hat{a}_y + \omega_z \hat{a}_z^\dagger \hat{a}_z + \sum_{\alpha=x,y,z} \sum_{i=1}^{4} g_i^\alpha (\hat{a}_i^\dagger \hat{a}_\alpha + \hat{a}_\alpha^\dagger \hat{a}_i) + U_J(\hat{\phi}_x, \hat{\phi}_y, \hat{\phi}_z). \quad (26)$$

In the above expression, $\hat{a}_\alpha$ are the JRM mode operators and in terms of the zero point flux fluctuation, $\hat{\phi}_\alpha = \phi_\alpha(\hat{a}_\alpha + \hat{a}_\alpha^\dagger)$. The coupling rate is $g_i^\alpha = e^2\phi_0^2/(2C\phi_\alpha\phi_i)$. The potential U_J represents the interaction between the JRM modes and is expressed as

$$U_J = -E_J \left[2\cos\left(\frac{\phi_x - \phi_y}{2\phi_0}\right)\cos\left(\frac{\phi_z}{\phi_0} + 3\Phi_{ex}\right) + 2\cos\left(\frac{\phi_x + \phi_y}{2\phi}\right)\cos\left(-\frac{\phi_z}{\phi_0} + \Phi_{ex}\right)\right], \quad (27)$$

where $3\Phi_{\rm ex}$ and $\Phi_{\rm ext}$ are the unit-less external flux applied to the big and small loops respectively of the JRM. Note that one should subtract the second order term of each mode from the potential but, for simplicity, we omit these terms here because the correction to the frequencies are small. We now choose the external flux to be $\Phi_{ex} = \pi/2$ such that

$$U_J = -4E_J \cos\left(\frac{\phi_x}{2\phi_0}\right)\cos\left(\frac{\phi_y}{2\phi_0}\right)\sin\left(\frac{\phi_z}{\phi_0}\right). \quad (28)$$

The above expression for U_J is substituted in Eq. (26) to obtain a complete expression for the coupling Hamiltonian.

It is now possible to provide an overview for the generation of a tunable coupling between the JPA modes: the frequencies are designed such that the JPA modes are dispersively coupled only to the x-modes, and a classical drive of frequency $\omega_{\rm d}$ on the far detuned z-mode triggers the four-body coupling. The z-mode is driven by applying fields with equal strength, but opposite phases to the capacitors C_x and C_y as indicated by the dark and light arrows in Supplementary Figure **8**. Since there is no drive to the y-mode, it can be dropped from the Hamiltonian. As a result, the total Hamiltonian can be written as

$$\hat{H} = \sum_{k=1}^{4} \left[\hat{H}_{{\rm JPA},k} + g_k^x(\hat{a}_l^\dagger \hat{a}_x + \hat{a}_x^\dagger \hat{a}_k) + g_k^z(\hat{a}_k^\dagger \hat{a}_z + \hat{a}_z^\dagger \hat{a}_k)\right] + E_J \frac{\hat{\phi}_x^2}{2\phi_0^3}\hat{\phi}_z - E_J \frac{\hat{\phi}_x^4}{96\phi_0^5}\hat{\phi}_z. \quad (29)$$

Furthermore, if $\Delta_{\rm k} = \omega_x - \omega_{{\rm r},k} \gg g_k$, then it is possible to apply a dispersive unitary transformation $\hat{U} = \exp[-i(g_1/\Delta_1)\hat{a}_x^\dagger \hat{a}_1 - i(g_2/\Delta_2)\hat{a}_x^\dagger \hat{a}_2 + i(g_3/\Delta_3)\hat{a}_x^\dagger \hat{a}_3 + i(g_4/\Delta_4)\hat{a}_x^\dagger \hat{a}_4 + {\rm h.c.}]$ to the total Hamiltonian. As mentioned before, the z-mode is driven classically by a field of frequency $\omega_{\rm d}$ and we therefore replace $\hat{\phi}_z$ by the classical amplitude $2\phi_z\sqrt{n}\cos(\omega_{\rm d}t)$, where n is the number of photons in the mode z. If $\omega_{\rm d} = \omega_{{\rm p},1} + \omega_{{\rm p},2} + \omega_{{\rm p},3} - \omega_{{\rm p},4}$ then it is possible to eliminate the fast rotating terms and the resulting Hamiltonian is

$$\hat{H}_{\rm plaquette} \approx \sum_{k=1}^{4} \left(\hat{H}_{{\rm JPA},k} - \frac{(g_k^x)^2}{\Delta_k^x}\hat{a}_k^\dagger \hat{a}_k \right) - C_{\rm jrm}(\hat{a}_1^\dagger \hat{a}_2^\dagger \hat{a}_3^\dagger \hat{a}_4 + {\rm h.c.}), \quad (30)$$

where

$$C_{\rm jrm} = E_J\sqrt{n}\frac{\phi_x^4\phi_z}{4\phi_0^5}\frac{g_1g_2g_3g_4}{\Delta_1\Delta_2\Delta_3\Delta_4}. \quad (31)$$

Note that, in the computational basis, the interaction term $C_{\rm jrm}(\hat{a}_1^\dagger \hat{a}_2^\dagger \hat{a}_3^\dagger \hat{a}_4 + {\rm h.c.})$ can be expressed as $2C_{\rm jrm}{\rm Re}[\alpha_0^3\alpha_0^*]\hat{\sigma}_{{\rm z},1}\hat{\sigma}_{{\rm z},2}\hat{\sigma}_{{\rm z},1}\hat{\sigma}_{{\rm z},3}\hat{\sigma}_{{\rm z},4}$, which is the desired four-body coupling. By taking $E_J/2\pi = 860$ GHz, $g_k/\Delta_k = 0.12$, $\phi_x/\phi_0 = \phi_z/\phi_0 = 0.12$ and $n = 2.25$, it is possible to obtain a four-body coupling strength $C_{\rm jrm}/2\pi = 1.7$ KHz. Note that this is a higher-order coupling compared with the fixed one analyzed in Supplementary Note 4 and therefore is an order of magnitude smaller. The advantage of this scheme, however, is that it is tunable and that the coupling strength can be increased by increasing $\sqrt{n}$, which is proportional to the strength of the applied microwave drive. The coupling could also be increased by increasing the zero-point flux fluctuations ϕ_x and ϕ_z.

SUPPLEMENTARY REFERENCES

[1] Puri, S., Boutin, S. & Blais, A. Engineering the quantum states of light in a kerr-nonlinear resonator by two-photon driving. *npj Quantum Information* **3**, 18 (2017).

[2] Johansson, J., Nation, P. & Nori, F. Qutip: An open-source python framework for the dynamics of open quantum systems. *Computer Physics Communications* **183**, 1760–1772 (2012).

[3] Johansson, J., Nation, P. & Nori, F. Qutip 2: A python framework for the dynamics of open quantum systems. *Computer Physics Communications* **184**, 1234–1240 (2013).

[4] Lechner, W., Hauke, P. & Zoller, P. A quantum annealing architecture with all-to-all connectivity from local interactions. *Science Advances* **1**, e1500838 (2015).

[5] Bourassa, J., Beaudoin, F., Gambetta, J. M. & Blais, A. Josephson-junction-embedded transmission-line resonators: From kerr medium to in-line transmon. *Physical Review A* **86**, 013814 (2012).

[6] Wustmann, W. & Shumeiko, V. Parametric resonance in tunable superconducting cavities. *Physical Review B* **87**, 184501 (2013).

[7] Mirrahimi, M. *et al.* Dynamically protected cat-qubits: a new paradigm for universal quantum computation. *New Journal of Physics* **16**, 045014 (2014).
